# Influence of Varying Pre-Culture Conditions on the Level of Population Heterogeneity in Batch Cultures with an *Escherichia coli* Triple Reporter Strain

**DOI:** 10.3390/microorganisms11071763

**Published:** 2023-07-06

**Authors:** Manh Dat Hoang, Sophi Riessner, Jose Enrique Oropeza Vargas, Nikolas von den Eichen, Anna-Lena Heins

**Affiliations:** Chair of Biochemical Engineering, TUM School of Engineering and Design, Technical University of Munich, 85748 Garching, Germany; dat.hoang@tum.de (M.D.H.); ga87bok@mytum.de (S.R.); enrique.oropeza@tum.de (J.E.O.V.); nikolas.eichen@tum.de (N.v.d.E.)

**Keywords:** pre-culture, population heterogeneity, triple reporter strain, batch culture, principal component analysis

## Abstract

When targeting robust, high-yielding bioprocesses, phenomena such as population heterogeneity have to be considered. Therefore, the influence of the conditions which the cells experience prior to the main culture should also be evaluated. Here, the influence of a pre-culture medium (complex vs. minimal medium), optical density for inoculation of the main culture (0.005, 0.02 and 0.0125) and harvest time points of the pre-culture in exponential growth phase (early, mid and late) on the level of population heterogeneity in batch cultures of the *Escherichia coli* triple reporter strain G7_BL21(DE3)_ in stirred-tank bioreactors was studied. This strain allows monitoring the growth (*rrnB*-EmGFP), general stress response (*rpoS*-mStrawberry) and oxygen limitation (*nar*-TagRFP657) of single cells through the expression of fluorescent proteins. Data from batch cultivations with varying pre-culture conditions were analysed with principal component analysis. According to fluorescence data, the pre-culture medium had the largest impact on population heterogeneities during the bioprocess. While a minimal medium as a pre-culture medium elevated the differences in cellular growth behaviour in the subsequent batch process, a complex medium increased the general stress response and led to a higher population heterogeneity. The latter was promoted by an early harvest of the cells with low inoculation density. Seemingly, *nar*-operon expression acted independently of the pre-culture conditions.

## 1. Introduction

Industrial bioprocesses should be robust and high-yielding to account as profitable. This implies the application of well-characterised production hosts, because phenomena such as population heterogeneity can lead to a significant alteration of host physiology [1]. Population heterogeneity captions the unequal behaviour of isogenic populations reacting to dynamically changing environmental conditions on their individual path inside the bioreactor [2]. These variable conditions result from mixing insufficiencies that promote the abundance of gradients of process state variables and thereby the potential emergence of different co-existing phenotypes [3]. Consequences can be a yield reduction and an increased by-product formation compared to laboratory-scale bioprocesses [4,5].

Population heterogeneity in bioprocesses can be studied by applying reporter strains [6,7]. They express one or several fluorescent proteins, whose pattern can be correlated to cellular characteristics of interest [8], for instance, growth [9,10,11], stress response [12,13,14] or product formation [15,16]. Combining several markers for different cellular characteristics in one strain significantly increases the level of information gained compared to employing single reporter strains [11,17]. The fluorescence of reporter strains in bioprocesses is predominantly analysed with *at-line* flow cytometry [1,18], which allows us to collect multivariate fluorescence data with temporal resolution for thousands of cells. To objectively quantify the intensity and shape variations of fluorescence distributions, a combination of simple descriptive parameters can be evaluated [19] such as mean and mode fluorescence and the distribution width. Furthermore, the coefficient of variance (CV) provides information about the heterogeneity level of a distribution [20,21]. Another distribution shape-related parameter is the skew that describes whether a distribution is tailing towards higher or lower fluorescence intensities [19].

To gain a complete picture of population heterogeneity in a bioprocess, the influence of the culture conditions which the cells are exposed to prior to the main culture should also be considered. Earlier studies investigated how the carbon source and the type of medium of the pre-culture, respectively, the size of inoculum, influences cellular growth, morphology and amongst others, the production of antibody fragments or cellulose during subsequent cultivation in shake flasks or bioreactors [22,23,24,25]. Optimizing the pre-culture conditions based on the experimental results generally led to shortened lag-phases and an improved overall process performance [26,27].

Since this has, to our knowledge, never been evaluated, our goal was to study the influence of pre-culture conditions concerning a medium in the pre-culture, optical density at inoculation of the main culture and harvest time of the pre-culture in exponential growth phase on single-cell physiology and the level of population heterogeneity in batch cultures with an *Escherichia coli* triple reporter strain G7_BL21(DE3)_ [11]. This strain allows us to follow single-cell growth by expression of EmGFP from the ribosomal promoter *rrnB*, the general stress response of single cells by expression of mStrawberry, along with the sigma factor *rpoS* and oxygen limitation of single cells expressing the *nar*-operon together with TagRFP657. The *nar-*operon codes for a subunit of a nitrate reductase that enables *E. coli* to metabolise nitrate as an electron acceptor under anoxic conditions [28]. In a recent study, however, it was shown that the *narGHIJ* operon in an *Escherichia coli* strain was already activated and expressed during stirred-tank bioreactor cultivations with dissolved oxygen levels between 20 and 40% [17]. Data analysis was performed with principal component analysis (PCA). This method was similarly applied earlier for the identification of the sources of undesirable process variabilities during cell culture manufacturing [29].

Lately, chemometric tools are increasingly applied for the classification of bioprocess data and the monitoring of process performance [29,30]. These multivariate mathematical approaches are powerful for a simplified representation of underlying similarities between samples that originate from complex analytical data [30]. Principal component analysis (PCA) is a popular tool for exploratory data analysis in various research [31,32,33]. It uncovers the pattern of subgroups within a dataset with a higher degree of similarity than the remaining data that are not part of this subgroup, and is often applied to reduce the dimensionality of large datasets and consequently the number of variables via a transformation process into smaller datasets, all while preserving as much information of the original dataset as possible [34]. PCA relies on orthogonal transformation through a bilinear decomposition of possibly correlated original variables into fewer linearly uncorrelated variables. These principal components (PCs) capture the largest variance in the data. During data transformation, the original data matrix is decomposed into a score and a loading matrix as well as a matrix of residuals. The loadings define the weights of the original variables on each PC. The scores represent the new variables [31,32,34]. As a preparation of the dataset prior to the actual analysis, the scale of the initial values is standardised to prevent the large-fold changes of a variable from dominating the analysis. Consequently, all variables are transformed to the same scale. As a next step, the covariance between the variables is computed (collected in a matrix) to determine whether some of the variables are redundant due to close correlation. Afterwards, the eigenvectors of the covariance matrix are computed and then ordered by their eigenvalues in descending order to identify the principal components and order them by significance. Principal components are the new variables that are constructed as the linear combinations of the initial variables. Some of their characteristics are that they are uncorrelated and that the first principal components account for most of the information about their corresponding variations in the original dataset, which then lead to a reduction in data dimensionality. To be able to plot the original data points as scores and loadings, the original data are transposed into values represented by the scale of the principal components.

**Figure 1:**
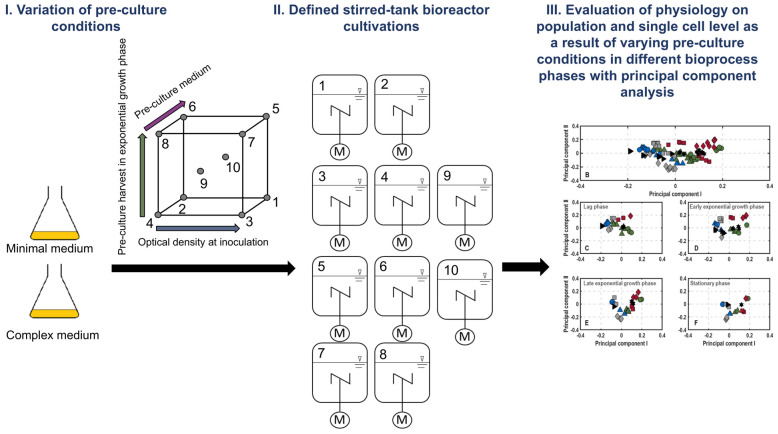
Overview of the experimental setup, investigating the impact of pre-culture conditions (pre-culture medium, optical density at inoculation and harvest time) on the level of population heterogeneity in subsequent stirred-tank bioreactor cultivations in defined medium at laboratory scale. Cellular physiologies were monitored by the corresponding reporter molecules and evaluated with principal component analysis.

A brief overview of the experimental setup used in this study is summarised in Figure 1. Additionally, the concept of reporter strains for the monitoring of cellular physiologies is depicted in the Supplementary Materials (see Supplementary Material III, Figure S5).

## 2. Materials and Methods

### 2.1. Triple Reporter Strain

The *Escherichia coli* BL21(DE3)-based triple reporter strain G7_BL21(DE3)_ expressing three chromosomally integrated fluorescent proteins, EmGFP, mStrawberry, and Tag-RFP657, respectively, which are connected to the expression of the ribosomal promoter *rrnB*, the promoter for expression of the sigma factor *rpoS*, and the *nar*-operon, respectively, was employed in this study. The triple reporter strain was synthesised in cooperation with Gen-H (Heidelberg, Germany), characterised in Heins et al. (2020) and stored as 15% glycerol stock at −80 °C [11].

### 2.2. Pre-Cultures

Single colonies from minimal medium [35] (MM) or complex Lennox lysogeny broth (LB) agar plates of G7_BL21(DE3)_ were used to inoculate 50 mL MM or LB cultures in 500 mL baffled shake flasks. They were shaken at 150 rpm and 37 °C for 6 h, 7 h or 9 h (MM), respectively, and 3.5 h, 5 h and 7 h (LB), corresponding to early, mid and late exponential growth phases. These time points were chosen based on results from preliminary shake flask cultures (see Supplementary Material I, Figures S1–S3). Stirred-tank bioreactors were then inoculated with the volume necessary to achieve an optical density at 600 nm (OD_600_) of 0.005, 0.0125 or 0.02. An overview of the experimental design is summarised in Table 1. Note that the harvest time point at mid-exponential growth phase with mid-optical density for inoculation in both media was chosen as centre points of the experimental design (Figure 1).

### 2.3. Bioreactor Cultures

Batch processes were performed in a 1.5 L stirred-tank bioreactor (Labfors 5, Infors AG, Bottmingen, Switzerland) with a working volume of 1.2 L. Cells were cultured in MM [35] with an initial glucose concentration of 20 g L^−1^. Sensors for pH and dissolved oxygen (VisiFerm DO ECS, Hamilton Bonaduz AG, Bonaduz, Switzerland) were calibrated according to standard procedures using a two- and one-point calibration, respectively (pH 4.02 and pH 9.21, calibration for 100% dissolved oxygen). The pH was controlled at pH 6.8 with 1 M ammonia. Temperature and stirring were kept constant at 37 °C and 1000 rpm. Aeration was set to 2 vvm at inoculation and automatically increased stepwise to a maximum of 8 vvm to avoid oxygen limitation. Continuous analysis of off-gas carbon dioxide (CO_2_) was performed using a gas sensor (BlueInOne Ferm, BlueSens, Herten, Germany). Following the phases of a typical batch culture, samples for HPLC, dry cell weight (DW) measurement and flow cytometry (FC) analysis were frequently withdrawn. Samples for FC analysis and DW were directly analysed, whereas samples for HPLC analysis were centrifuged for 5 min at 16,438× *g*, filtrated (0.20 µm pore size) and kept at −20 °C until analysis.

### 2.4. Sample Analysis

Biomass determination: For biomass determination, 1 mL cell suspension was centrifuged for 5 min at 16,438× *g* in pre-dried (24 h at 80 °C), weighted 2 mL Eppendorf tubes. The supernatant was used for HPLC samples and the remaining pellet was dried for 24 h at 80 °C, cooled down to room temperature and weighted. DW was determined by the weight-difference between empty and filled tube.

Flow cytometry analysis: Cell suspension samples were centrifuged for 5 min at 16,438× *g*. The pellet was re-suspended and diluted in sterile 0.9% saline solution, equivalent of an optical density at 600 nm of about 0.1, and directly analysed. A CytoFLEX (Beckman Coulter, Brea, CA, USA) equipped with lasers at 488 nm and 638 nm was employed for analysis. The minimum laser power was 50 mW and the beam spot size 5 µm × 80 µm. The flow cytometer has nine detection channels for forward scatter (FSC), side scatter (SSC) and six fixed wavelengths (525/40 nm, 585/42 nm, 610/20 nm, 660/20 nm and 2× 780/60 nm). Fluorescence of the G7_BL21(DE3)_ was collected at 525/40 nm for EmGFP expression related to single-cell growth and at 585/42 nm for mStrawberry expression related to general stress response of single cells. Both fluorescent proteins were excited by the 488 nm laser. Fluorescence of TagRFP657 correlated to oxygen limitation of single cells was excited with the 638 nm laser and collected at 660/20 nm. To eliminate background noise, the detection threshold was set in SSC. In total, 1,000,000 events were recorded with a rate of around 1000 events per second. During analysis, fluorescence distributions were visualised with the FC proprietary software CytExpert. Obtained results were saved in FCS 3.0 format.

HPLC analysis: Concentrations of glucose, succinate, acetate, malate, lactate and formate were quantified by HPLC (Finnigan Surveyor, Thermo Fisher Scientific, Waltham, MA, USA). Detection was performed by refractive index (RID Agilent 1200, Agilent Technologies, CA, USA) with a 300 mm × 7.8 mm Aminex HPX-87H ion exchange column (Bio-Rad, Hercules, Santa Clara, CA, USA). An amount of 20 µL of sample was injected into the mobile phase of 5 mM H_2_SO_4_ (aq.) at a temperature of 60 °C and a flow rate of 0.35 mL min^−1^. Aside from generating time series plots, results from HPLC analysis and biomass determination were used to calculate yield coefficients and the carbon balance (see Supplementary Material II, Figure S4).

### 2.5. Data Analysis

Raw FCS 3.0 files were loaded into MATLAB^®^ R2017a (The MathWorks, Inc., Natick, MA, USA) using the function *fcsread* (by Robert Henson, available on MATLAB^®^ central file sharing). Then, the shape and intensity of fluorescence distributions was evaluated according to Heins et al. (2019) [19]. The MATLAB^®^ built-in function *mean*, was employed to calculate mean fluorescence. Furthermore, the fluorescence distribution width at baseline level was computed. Screening for fluorescence channels with at least five counts exceeding the number of random cell counts (3 ± 0.5) discriminated distributions from background noise. The distribution width was then determined by subtracting the channel numbers of the lowest channel with five counts and the respective highest channel. To account for variance in distributions, the coefficient of variance (CV) was determined by dividing distribution width by mean fluorescence. Skew of distributions was identified by subtracting the mode of distributions, which was generated utilizing the MATLAB^®^ built-in function *mode* from its mean. Positive values indicated a right skew, whereas negative values were returned for left skews. A combination of CV values, skews and the widths of the corresponding histograms was shown to be descriptive and sufficient to investigate distributions of single-cell characteristics and evaluate the presence of potential subpopulations without significant loss of information [19].

For unrevealing the main influence factors of varying pre-culture conditions, principal component analysis was performed with physiology data on population level (concentration of biomass, glucose, acetate and formate as well as offgas data) and single-cell level (mean, CV, width, skew and mode of the fluorescence distributions of the three markers of the reporter strain). First, data were saved in a matrix listing the respective parameters for all process samples. Then, the matrix was pre-processed, averaging and interpolating data. Furthermore, pareto scaling was used with the square root of the standard deviation as scaling factor, to decrease the dominance of large fold changes. Afterwards, single value decomposition was performed using the MATLAB^®^ built-in function *svd*, generating unique coordinates for each sample. For score and loadings for the first two columns of the respective matrices covering the majority of variation in the dataset, scatter plots were generated. Samples from lag-phase, early exponential growth phase (samples in which the glucose concentration was above 15 g L^−1^ and by-product formation takes place), late exponential growth phase (samples in which the glucose concentration was below 15 g L^−1^ and re-assimilation of the by-products takes place) and stationary phase were plotted separately in subplots.

## 3. Results

### 3.1. Population-Level Physiology

**Figure 2:**
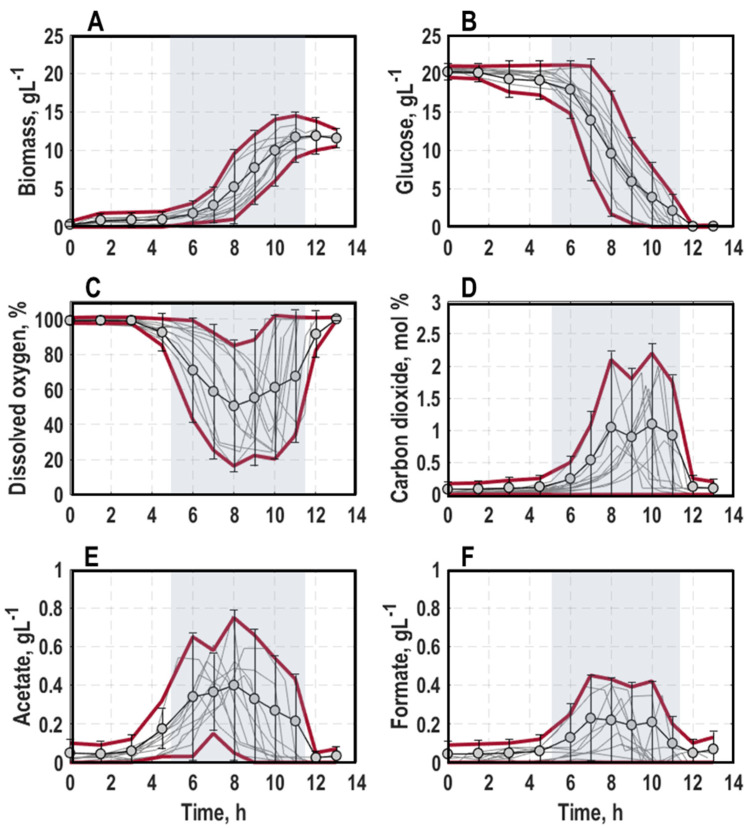
General trend of biomass (**A**) and glucose concentration (**B**), dissolved oxygen level in the bioreactor (**C**) and offgas carbon dioxide (**D**) as well as concentration of the by-products acetate (**E**) and formate (**F**) in batch cultivations of *E. coli* G7_BL21(DE3)_ in a stirred-tank bioreactor in minimal medium with glucose as carbon source varying optical density at 600 nm for inoculation (0.005, 0.02 or 0.0125), harvest time point of the pre-culture in exponential growth phase (early, mid or late) and pre-culture medium (lysogeny broth (LB) and minimal medium (MM) [35]). The grey background marks the average exponential growth phase of all processes.

General trend: The ten performed batch cultivations exhibited typical growth behaviour of *E. coli* batch cultures (Figure 2, for a detailed course of individual processes, consult Supplementary Material IV). First, the cultures exhibited a lag-phase of on average 5.5 ± 1.3 h with no obvious increase in biomass concentration, offgas carbon dioxide (CO_2_) and metabolites (Figure 2A,D–F). Additionally, the marginal consumption of glucose and dissolved oxygen in the bioreactor (DO) was found (Figure 2B,C). Then, cultures passed through a transition phase of 0.75 ± 0.35 h with a slight growth, minor formation of acetate and CO_2_ as well as a minor consumption of glucose and dissolved oxygen (DO) (Figure 2A–E). Thereafter, they entered exponential growth phase with an average maximum specific growth rate of all cultivations of 0.729 ± 0.009 h^−1^, which is consistent with an earlier study [11]. Meanwhile, glucose and DO were consumed (Figure 2B,C, grey background illustrates the mean exponential growth phase of all cultures). Concurrently, CO_2_ and the concentration of formate and acetate rose constantly, until around mid-exponential growth phase (Figure 2D–F). At the end of the processes, both by-products were simultaneously re-assimilated after the depletion of glucose in the bioreactor (Figure 2B,E,F). This process phase was also accompanied by a steep increase in DO and decrease in CO_2_-level (Figure 2C,D), and ultimately, the cultures entered stationary phase after around 11 ± 1.2 h of cultivation. Consistent with a previous study [11], an average final biomass concentration of 11.19 ± 1.35 g L^−1^ was reached.

As expected, yield coefficients and carbon balances, which closed between 96 and 106%, showed no significant variations for major metabolites (see Supplementary Material II, Figure S4) and matched the results from a previous study [11].

**Figure 3:**
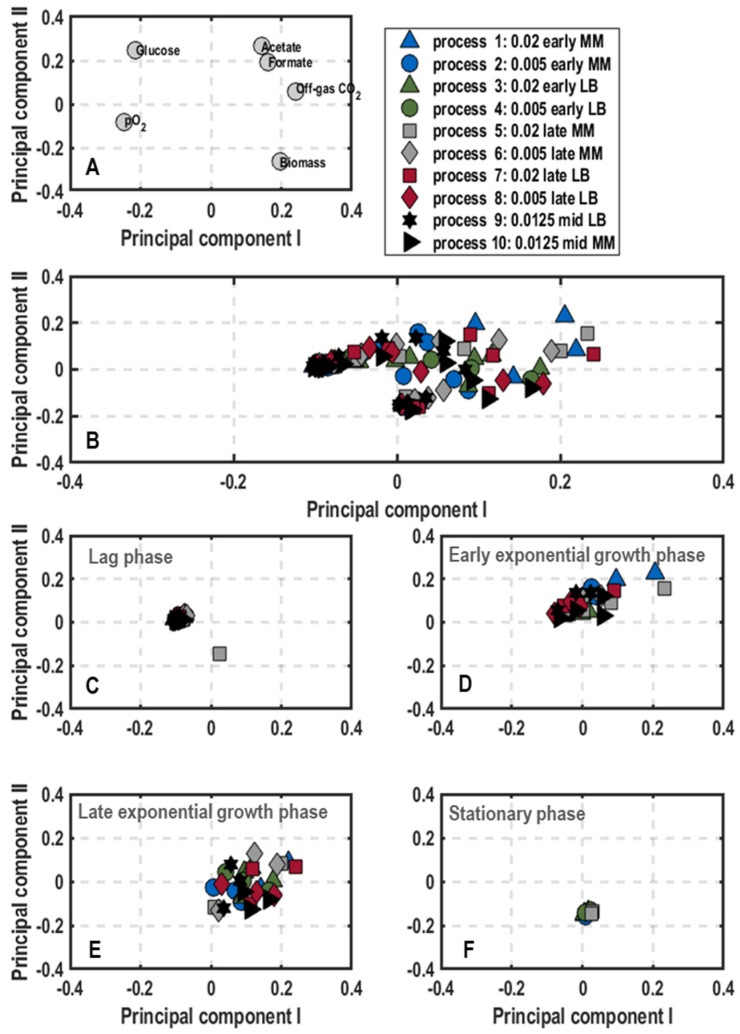
Plots of loadings (**A**) and score plots (**B**–**F**) for the first and second principal component of population-level physiology samples including the concentrations of biomass, glucose, and the by-products acetate and formate as well as dissolved oxygen and offgas carbon dioxide in batch cultivations of *E. coli* G7_BL21(DE3)_ in stirred-tank bioreactors in minimal medium with glucose as carbon source varying optical density at 600 nm for inoculation (0.005, 0.02 or 0.0125), harvest time point of the pre-culture in exponential growth phase (early, mid or late) and pre-culture medium (lysogeny broth (LB) and minimal medium (MM) [35]). The overall score plot of the processes was subdivided into score plots with samples at different stages of the bioprocess (lag-phase (**C**), early (**D**) and late exponential phas€(**E**) and stationary phase (**F**)).

Cluster analysis: The results of the principal component analysis on population-level physiology data are shown as plots of scores and loadings of the first principal component (PC_1_) and the second principal component (PC_2_) (Figure 3) explaining 85.64% of the variance in the data. The loadings scatter in a semicircle in the upperpart of the plot (Figure 3A). The left lower end builds the DO_,_ followed by the concentrations of glucose, acetate and formate at the top. On the right side, the CO_2_ scatters above the biomass concentration. Hence, PC_1_ separates lag-phase samples with high glucose concentration (values of PC_1_ negative and PC_2_ positive) and high DO values (values of PC_1_ negative and PC_2_ neutral) from stationary phase samples with high values of biomass and CO_2_ and by-product formation (positive values of PC_1_) (Figure 2 and Figure 3). In contrast, higher PC_2_ values indicate a stronger by-product formation, while negative values refer to high biomass production. Hence, the cultivations can be followed along PC_1_, whereas differences in metabolism are illustrated by PC_2_. Based on this finding, besides plotting the data of different process stages of all ten performed batch processes (Figure 3B), further clustering showing samples of the initial lag-phase, the early and late exponential growth phase as well as the stationary phase separately was performed (Figure 3C–F).

According to the score plot of the initial lag-phase (Figure 3C), the samples of all processes showed slightly negative PC_1_ values and a neutral PC_2_ with exception of process 5 (0.02/late/MM), which was shifted towards the lower right corner in comparison to the other samples. This translates to high glucose concentrations and dissolved oxygen levels, whereas biomass production and by-product formation (CO_2_, formate and acetate) were not yet initiated. Despite the different harvesting conditions from the pre-culture, no differences were visible (Figure 3C). As a general trend in early exponential growth phase (Figure 3D), samples of all processes moved towards the upper right corner with positive PC_2_ values. Interestingly, samples of the processes inoculated with pre-cultures grown in complex medium showed less metabolic activity (processes 3, 4, 7, 8 and 9, negative PC_1_) in comparison to processes, which were inoculated with pre-cultures grown in minimal medium (processes 1, 2, 5, 6, 10, positive PC_1_). This was particularly applicable for the processes 1 (0.02/early/MM) and 5 (0.02/late/MM) with PC_2_ values of around 0.2 and thus the highest by-product formation at this process stage (Figure 3D). Heading forward to the late exponential growth phase (Figure 3E), samples in the score plot all transitioned to the right side with positive PC_1_ values, but were scattered between positive and negative PC_2_ values. Process 5 (0.02/late/MM), 6 (0.005/late/MM) and 7 (0.02/late/LB) tended towards positive PC_2_ values representing high carbon dioxide evolution rates and by-product formation with low biomass amount. Consequently, these processes just reached the maximum growth rate and started to produce biomass. On the other hand, processes 2 (0.005/early/MM) and 10 (0.0125/mid/MM) settled on the negative side of PC_2_, thus probably already passed the maximum growth rate phase and were on the verge to stationary phase (Figure 3E). Hence, the earlier the cells were harvested during preliminary cultivation in minimal medium, the faster they grew in subsequent batch processes in a stirred-tank bioreactor. For the stationary phase, samples of all processes accumulated at the bottom part of the score plot with neutral PC_1_ and negative PC_2_ values (Figure 3F).

### 3.2. Single-Cell Physiology

#### 3.2.1. Single-Cell Growth

**Figure 4:**
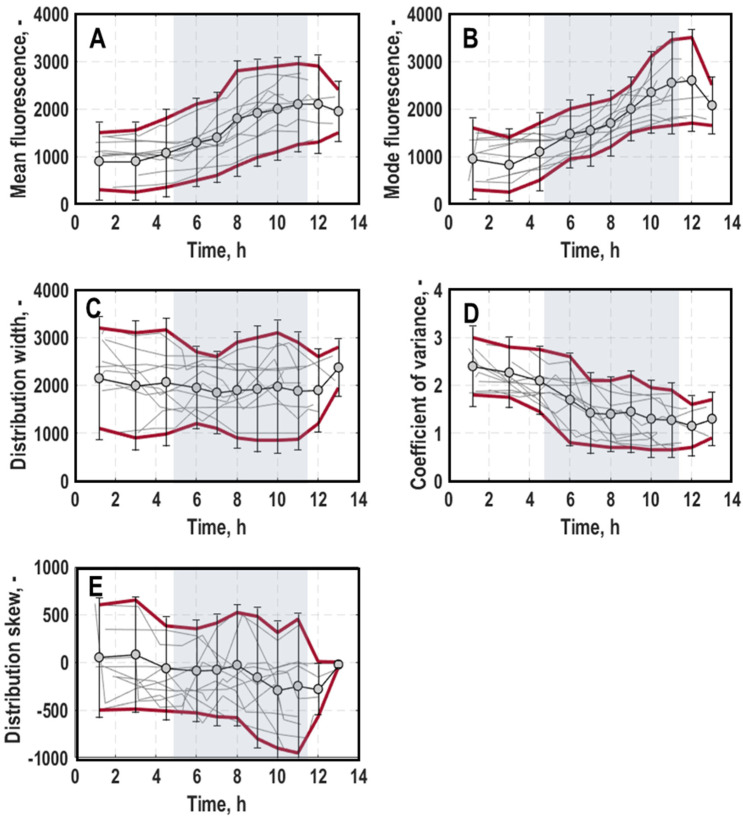
General trend of mean (**A**), mode (**B**), width (**C**), coefficient of variance (**D**) and skew (**E**) of distributions for single-cell growth (*rrnB-*EmGFP expression) measured at 525/40 nm in batch cultivations of *E. coli* G7_BL21(DE3)_ in stirred-tank bioreactors varying optical density at 600 nm for inoculation (0.005, 0.02 or 0.0125), harvest time point of the pre-culture in exponential growth phase (early, mid or late) and pre-culture medium ((lysogeny broth (LB) and minimal medium (MM) [35]). Grey background marks the average exponential growth phase of all processes. Data of all processes are averaged and the corresponding standard deviation is visualised by the red lines.

General trend: Generally, mean fluorescence for a growth-dependent expression of *rrnB*-EmGFP of all cultures mirrored cellular growth on population level (Figure 2 and Figure 3A, for a detailed course of individual processes, consult Supplementary Material V), which is consistent with an earlier study [11]. It was constant during lag-phase, then rose until around mid-exponential growth phase, from whereon it stagnated, respectively, then gradually decreased until the end of the cultivations. Opposite to growth on the population level, cultivations that experienced different pre-culture conditions deviated stronger regarding expressed fluorescence intensities at 525/40 nm, especially at the beginning and end of the cultivation (differences of mean fluorescence values at process start: 1021.52 ± 338.35 and at process end: 2070.58 ± 467.51). The mode of distributions for single-cell growth (Figure 4B) was relatively uniform, especially during exponential growth phase. Other than that, its course resembled the mean fluorescence values. The distribution width (Figure 4C) did not show significant variation within cultivations apart from a slight decline during exponential growth phase but exhibited strong differences between different processes, especially during the initial lag-phase (standard deviation at 1520.28) and the late exponential growth between 8 and 11 h (standard deviation at 1290.23). The overall spread of all cultivations was at 2107.91 ± 564.20. The coefficient of variance (CV) (Figure 4D) exhibited an inverse correlation to mean fluorescence, which is consistent with the literature [20]. It exhibits values of around 2.18 ± 0.37 at the beginning and declined towards 1.07 ± 0.44 at the end of the cultivations. For some cultivations, however, the decline of the slope was less pronounced and almost linear, so that the CV was heterogeneous when comparing different cultivations. Also, the skew (Figure 4E) exhibited a strong spread between different cultures, again particularly at the late exponential growth phase between 9 and 11 h (standard deviation at 916.88). For most cultivations, a gradually increasing trend during exponential growth phase was seen, resulting in predominantly right-skewed and regular-shaped distributions in later process stages, respectively.

**Figure 5:**
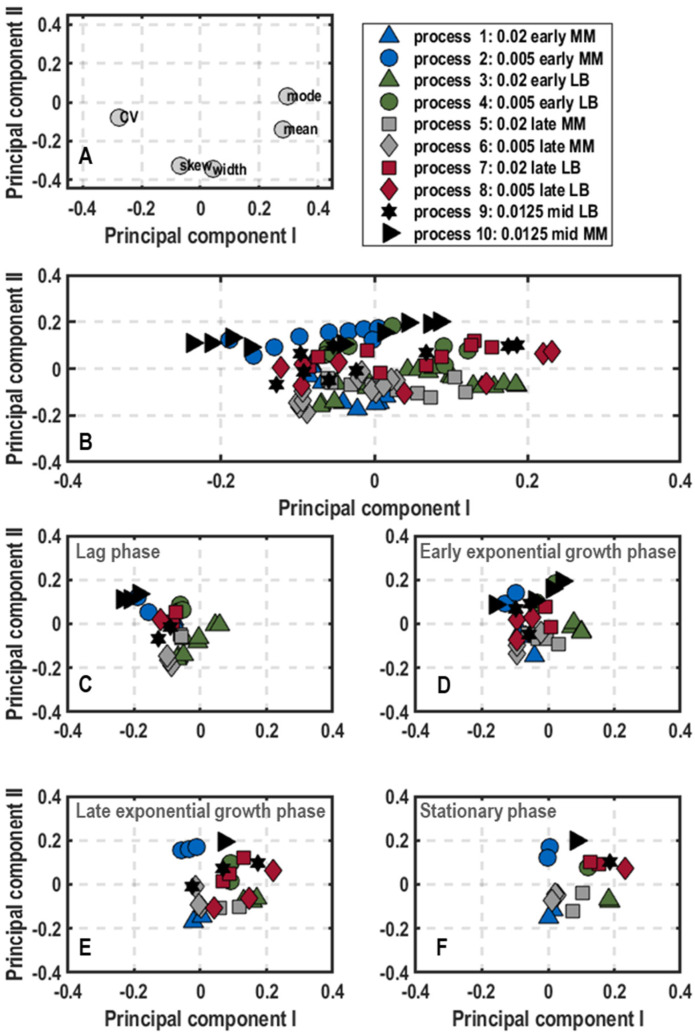
Plots of loadings (**A**) and score plots (**B**–**F**) for the first and second principal components of calculated parameters that describe the shape and intensity of distributions for single-cell growth (*rrnB*-EmGFP expression), including mean, coefficient of variance, mode, skew and width. Data originate from batch cultivations of *E. coli* G7_BL21(DE3)_ in stirred-tank bioreactors in minimal medium with glucose as carbon source varying optical density at 600 nm for inoculation of the bioreactor (0.005, 0.02 or 0.0125), harvest time point of the pre-culture in exponential growth phase (early, mid or late) and pre-culture medium (lysogeny broth (LB) and minimal medium (MM) [35]). The overall score plot of the processes was subdivided into score plots with samples at different stages of the bioprocess (lag-phase (**C**), early and late exponential phase (**D**,**E**) and stationary phase (**F**)).

Cluster analysis: Plots of scores and loadings of PC_1_ and PC_2_ (Figure 5) for single-cell growth explained 87.54% of the variance in the data. The loadings scatter in a semicircle in the lower part of the plot (Figure 5A). The left upper end builds the CV, followed by skew and width, and on the right side, the mean and mode. Consequently, PC_1_ separates samples with high CV value from samples with high mean and mode values, whereas skew and width only have minor influence on PC_1_. The mode has no influence on PC_2_, whereas the other parameters settle at negative PC_2_ values. Thus, skew and width exhibit stronger negative values than the mean and the CV.

At the beginning of the process, the measured fluorescence signals at 525/40 nm were rather characterised by a high CV than high mean or mode values. Logically, the growth of the cells did not start at that time so that almost all samples landed on the left side of the score plot (Figure 5C). Remarkably, for processes inoculated with pre-cultures derived from minimal media, growth showed either higher CV levels (process 2 (0.005/early/MM) and process 10 (0.0125/mid/MM)) or higher skew and width values (process 1 (0.02/early/MM), process 5 (0.02/late/MM) and process 6 (0.005/late/MM)) compared to other processes. On the other hand, process 3 (0.02/early/LB) exhibited neutral to positive PC_1_ values; therefore, it already showed comparably high mean and mode values, indicating a quick start in growth.

With the entrance into early exponential growth phase (Figure 5D), samples of all processes moved to the right towards higher mean and mode values, but most of the samples still had negative PC_1_ values. Only samples from process 3 (0.02/early/LB) had completely positive PC_1_ values. Furthermore, all other samples were mainly placed in a rectangle between PC_1_ and PC_2_ values, ranging from −0.2 to 0 and −0.2 to 0.2, respectively. In this area, process 2 (0.005/early/MM) and process 10 (0.0125/mid/MM) were again placed at the far-left edge (high CV), while samples of process 1 (0.02/early/MM), process 5 (0.02/late/MM) and process 6 (0.005/late/MM) stood out as the bottom of cluster (Figure 5D). Furthermore, through comparing bioprocesses that deviated by inoculation optical density, generally, the higher inoculated processes settled further right in the plot continuing the trend of the lag-phase. Moreover, while processes that were inoculated with older pre-cultures (process 5–8) exhibited marginal difference in PC_2_ values, this difference was more pronounced for processes that were inoculated with fresh cells (process 1–4) or with mid-aged pre-cultures (process 9–10).

In late exponential growth phase, the sample pool continued to move from higher heterogeneity levels (skew and width) towards higher mean and mode values, now with the majority located at the right side of the score plot with positive PC_1_ values (Figure 5E). This trend was most pronounced in samples of process 4 (0.005/early/LB), process 7 (0.02/late/LB), process 8 (0.005/late/LB) and process 9 (0.0125/mid/LB), while samples of process 3 (0.02/early/LB) kept their high mean and mode profile. In contrast, samples of process 1 (0.02/early/MM), process 2 (0.005/early/MM) and process 6 (0.005/late/MM) remained at neutral PC_1_ values. Regarding population heterogeneity levels, processes in which the pre-cultures were grown in minimal media once again displayed the highest skew and widths of the measured histograms. In particular, process 1 (0.02/early/MM), process 5 (0.02/late/MM) and process 6 (0.005/late/MM) had the lowest PC_2_ values. Additionally, process 8 (0.005/late/LB) showed tendencies for skewed histograms with a broad width.

All the trends described for this phase were applicable for the final stationary phase, likely due to the fact that the cells in all the processes did not further grow at the end (Figure 5F).

#### 3.2.2. General Stress Response of Single Cells

**Figure 6:**
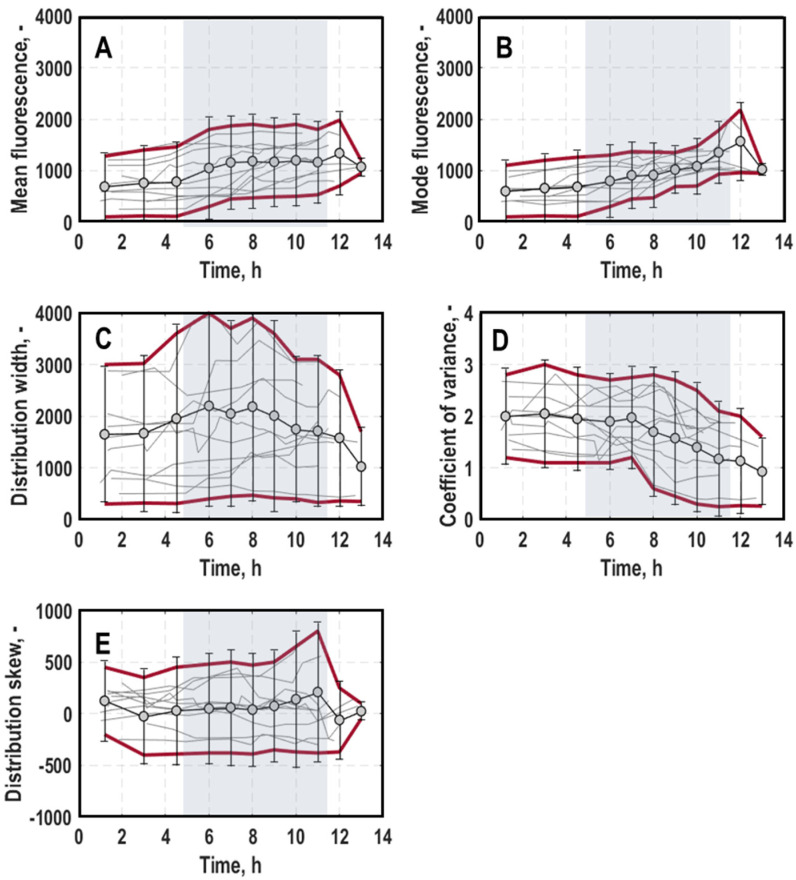
General trend of mean (**A**), mode (**B**), width (**C**), coefficient of variance (**D**) and skew (**E**) of distributions for the general stress response of single cells (*rpoS-*mStrawberry expression) in batch cultivations of *E. coli* G7_BL21(DE3)_ in stirred-tank bioreactors varying optical density at 600 nm for inoculation of the bioreactor (0.005, 0.02 or 0.0125), harvest time point of the pre-culture in exponential growth phase (early, mid or late) and pre-culture medium (lysogeny broth (LB) and minimal medium (MM) [35]). Grey background marks the average exponential growth phase of all processes. Data of all processes are averaged and the corresponding standard deviation is visualised by the red lines.

General trend: Generally, the mean and mode fluorescence of distributions for *rpoS*-mStrawberry were constant at the beginning of the cultivation (Figure 6A,B, for a detailed course of individual processes, consult SSupplementary Material VI) but at different initial levels. During the transition to exponential growth phase, mean and mode linearly increased for some cultivations. For the most processes, however, a distinct rise in fluorescence of about 34% was first seen from mid-exponential growth phase onwards. Afterwards, mean and mode remained relatively constant until the cultivation ended. The rise in fluorescence levels in exponential growth phase was expected by the characteristics of *rpoS*, which becomes active, among other things, when cells encounter high cell density in combination with low nutrient availability [11,17,36]. For most processes, the distribution width (Figure 6C) increased slightly at the onset of exponential growth phase. Otherwise, it remained constant but still with a strong spread of 1946.93 ± 868.85 between different processes. The CV (Figure 6D) was generally the highest at the beginning of the cultivations but decreased with the rise in mean and mode values. It exhibited a large spread of 1.67 ± 0.56 between different cultivations, pointing towards various levels of population heterogeneity. The skew (Figure 6E) remained constant for most processes around zero, which points towards almost Gaussian-shaped distributions on average. Nevertheless, some processes (mainly process 2, 4, 8, 9 and 10) exhibited left- and right-skewed distributions with increasing right-skew values during exponential growth phase with a standard deviation of 834.39.

**Figure 7:**
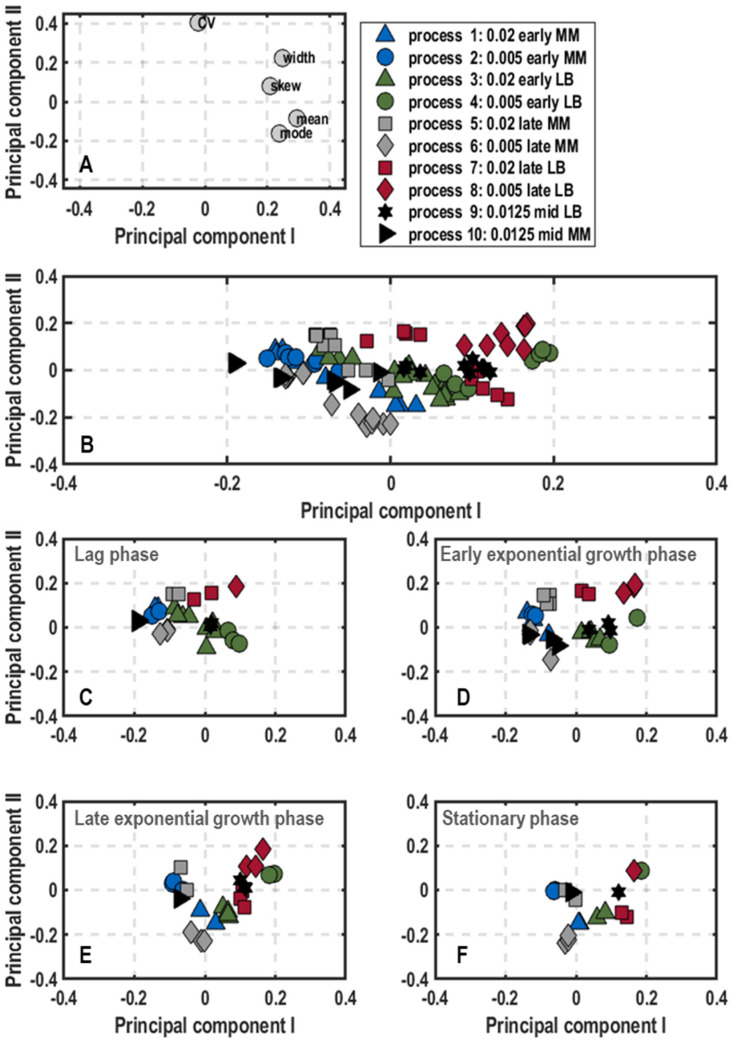
Plots of loadings (**A**) and score plots (**B**–**F**) for the first and second principal components of calculated parameters that describe the shape and intensity of distributions for general stress response of single cells (*rpoS*-mStrawberry-expression), including mean, coefficient of variance, mode, skew and width. Data originate from batch cultivations of *E. coli* G7_BL21(DE3)_ in stirred-tank bioreactors in minimal medium with glucose as carbon source varying optical density at 600 nm for inoculation of the bioreactor (0.005, 0.02 or 0.0125), harvest time point of the pre-culture in exponential phase (early, mid or late) and pre-culture medium (lysogeny broth (LB) and minimal medium (MM) [35]). The overall score plot of the processes was subdivided into score plots with samples at different stages of the process (lag-phase (**C**), early and late exponential phase (**D**,**E**) and stationary phase (**F**)).

Cluster analysis: Plots of scores and loadings of PC_1_ and PC_2_ (Figure 7) for the general stress response of single cells explained 73.33% of the variance in the data. According to the loading plot, PC_1_ unravels high and low values of all parameters but the CV, which has no influence on PC_1_ (Figure 7A). PC_2_ separates samples with peculiar mean and mode values (negative values) from samples that exhibit higher CV values, broader widths and stronger skew (positive values).

For this marker, a general trend can be anticipated when evaluating the score plot of all samples of the processes. Samples of processes which were inoculated with pre-cultures grown in complex medium were located on the right-hand side (positive PC_1_ values, scattered PC_2_ values), whereas samples of processes with pre-cultures grown in minimal media were more orientated on the left side of the score plot (negative PC_1_ values, scattered PC_2_ values) (Figure 7B).

When focussing on the single phases of the processes (Figure 7C), almost all lag-phase samples had negative PC_1_ values, and thus were positioned on the left side of the score plot. Only samples of process 4 (0.005/early/LB) and process 8 (0.005/late/LB) clearly exhibited positive PC_1_ values with a corresponding negative PC_2_ value for the former (higher stress levels) and a positive PC_2_ value for the latter process (higher population heterogeneity). When comparing process 5 (0.02/late/MM) and process 6 (0.005/late/MM), which only differ regarding the inoculation density, data points of the former were on the positive side of the PC_2_ values while data points of the latter were slightly negative. The direct comparison between process 9 (0.0125/mid/LB) and process 10 (0.0125/mid/MM) indicates further effects of the pre-culture media. While cells derived from complex medium out of the pre-culture showed neutral PC_1_ values, cells derived from minimal medium had rather negative PC_1_ values of around −0.2 (Figure 7C).

In early exponential growth phase, all samples of processes inoculated with pre-cultures grown in complex medium moved to the right side of the plot, thus exhibiting positive PC_1_ values. Here, samples of process 7 (0.02/late/LB) and process 8 (0.005/late/LB) were more pronounced at high CV and width, while single-cell stress response levels in process 3 (0.02/early/LB), process 4 (0.005/early/LB) and process 9 (0.0125/mid/LB) were rather characterised by high mean and mode values as well as higher skew values. In contrast, process 1 (0.02/early/MM), process 2 (0.005/early/MM), process 5 (0.02/late/MM), process 6 (0.005/late/MM) and process 10 (0.0125/mid/MM) did not score differently based on the previous phase and remained with negative PC_1_ values (Figure 7D).

Moving forward to the late exponential growth phase, processes inoculated with pre-cultures grown in minimal medium (process 1–2, 5–6 and 9–10) further moved to the right side of the score plot (neutral PC_1_ values). Simultaneously, process 1 (0.02/early/MM) and process 6 (0.005/late/MM) additionally exhibited stronger negative PC_2_ scores than in the previous phases. However, no clear trend can be recognised. For the processes inoculated with cells grown in a complex medium during pre-cultivation, it is striking that the cells in processes inoculated with lower cell densities (process 4 (0.005/early/LB) and process 8 (0.005/late/LB)) showed higher PC_1_ and PC_2_ values than processes inoculated with higher initial cell densities (process 3 (0.02/early/LB), process 7 (0.02/late/LB) and process 9 (0.0125/mid/LB)). These tendencies were also applicable for the stationary phase. Interestingly, the trends described at the beginning of the process between process 5 and 6 (differences in PC_2_ values) and process 9 and 10 (differences in PC_1_ values) remained valid in all phases (Figure 7E,F).

#### 3.2.3. Oxygen Limitation of Single Cells

**Figure 8:**
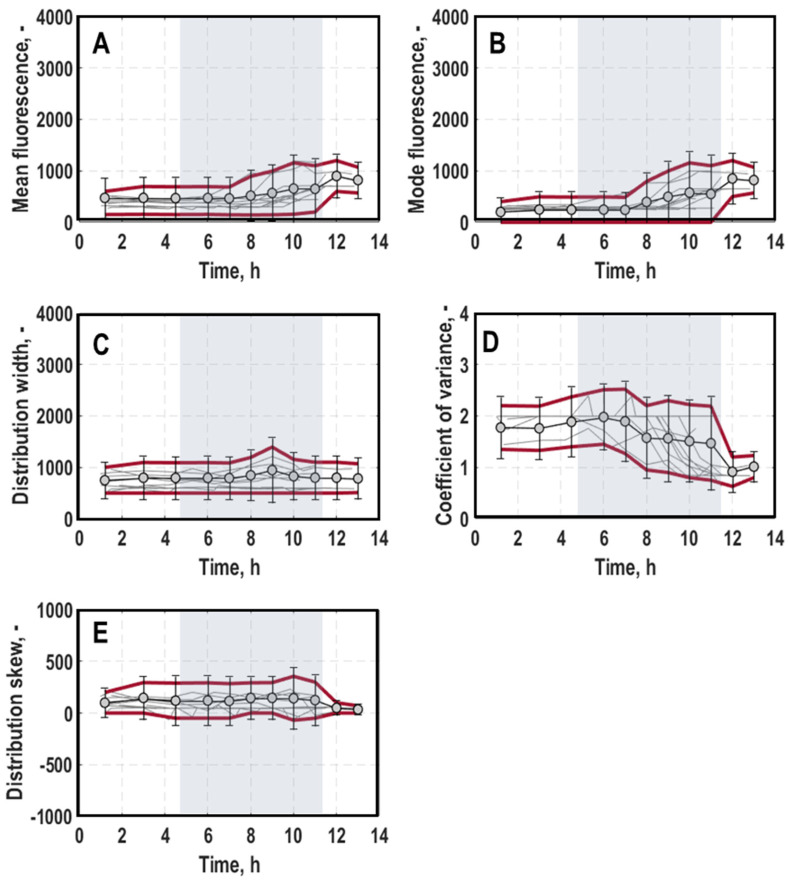
General trend of mean (**A**), mode (**B**), width (**C**), coefficient of variance (**D**) and skew (**E**) of distributions for oxygen limitation of single cells (*nar*-TagRFP657 expression) in batch cultivations of *E. coli* G7_BL21(DE3)_ in stirred-tank bioreactors varying optical density at 600 nm for inoculation of the bioreactor (0.005, 0.02 or 0.0125), harvest time point of the pre-culture in exponential phase (early, mid or late) and pre-culture medium (lysogeny broth (LB) and minimal medium (MM) [35]). Grey background marks the average exponential growth phase of all processes. Data of all processes are averaged and the corresponding standard deviation is visualised by the red lines.

General trend: Consistent with previous studies, the expression of *nar*-TagRFP657 revealed less variation and lower fluorescence intensities compared to the other markers of the triple reporter strain [11,17]. The reason is that the marker is solely active under oxygen-limited conditions [28]. In accordance with DO levels in the bioreactor being below 40% in mid-exponential growth phase (Figure 2, for a detailed course of individual processes, consult Supplementary Material VII), the mean and mode values of *nar*-Tag-RFP657 distributions of all processes (Figure 8A,B) rose by 57% on average. Otherwise, both parameters remained constant and homogenous when comparing different processes. The distribution width (Figure 8C) was constant for all processes, except for minor variations in mid-exponential growth phase. Also, the CV (Figure 8D) exhibited constant values for the majority of the processes until mid-exponential growth phase. According to the inverse correlation to the mean, the CV then declined and afterwards remained level until the process ended. Additionally, no significant skew of the distributions was detected (Figure 8E).

**Figure 9:**
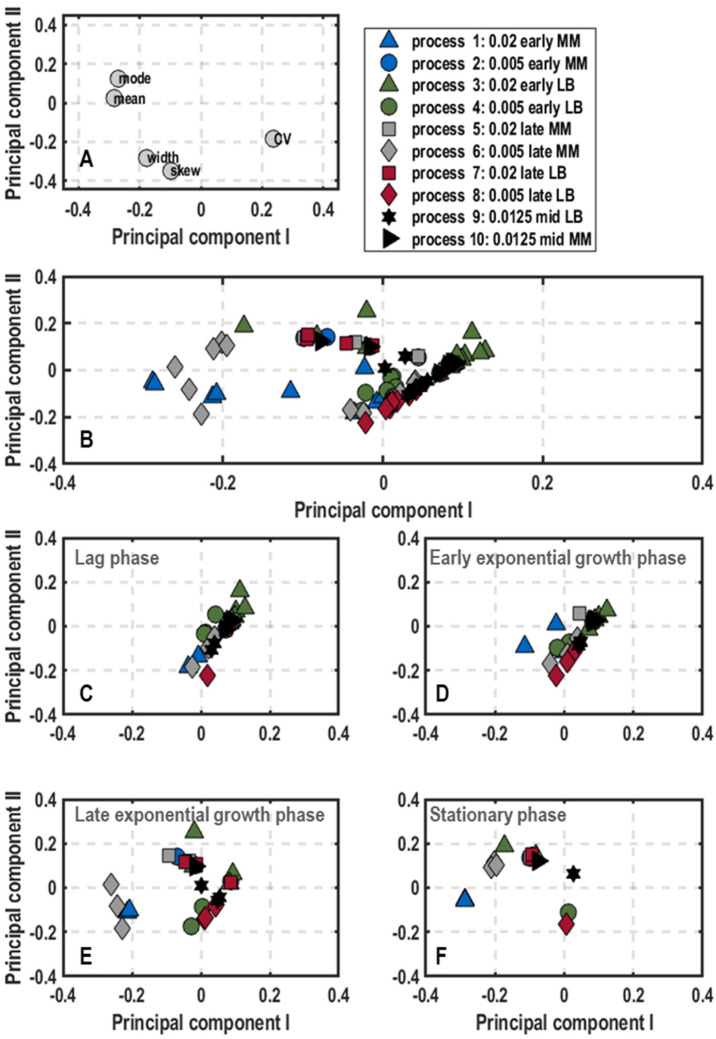
Plots of loadings (**A**) and score plots (**B**–**F**) for the first and second principal components of calculated parameters that describe the shape and intensity of distributions for oxygen limitation of single cells (*nar*-TagRFP657-expression**),** including mean, coefficient of variance, mode, skew and width. Data originate from batch cultivations of *E. coli* G7_BL21(DE3)_ in stirred-tank bioreactors in minimal medium with glucose as carbon source varying optical density at 600 nm for inoculation of the bioreactor (0.005, 0.02 or 0.0125), harvest time point of the pre-culture in exponential phase (early, mid or late) and pre-culture medium (lysogeny broth (LB) and minimal medium (MM) [35]). The overall score plot of the processes was subdivided into score plots with samples at different stages of the bioprocess (lag-phase (**C**), early and late exponential phase (**D**,**E**) and stationary phase (**F**)).

Cluster analysis: Plots of scores and loadings of PC_1_ and PC_2_ (Figure 9) for the oxygen limitation of single cells explained 77.71% of the variance in the data. PC_1_ separates the CV from the other parameters at positive values. Furthermore, skew and width settle at negative values, whereas mean and mode exhibited stronger negative values. PC_2_ isolates mean and mode at neutral-to-moderate positive values from the parameters that describe the distribution shape (skew, width and CV) (Figure 9A).

The score plot of all samples showed a few data points scattered on the left side, while a majority of sample points accumulated as a diagonal from neutral PC_1_ and slightly negative PC_2_ values towards the upper-right corner of the plot (Figure 9B). The latter data points had the largest distance to mean and mode and were present at the beginning lag-phase and the early exponential growth phase, indicating no oxygen limitation at these stages of the process (Figure 9C,D). This finding is consistent with the measured dissolved oxygen level in the bioreactor (Figure 2C). Nevertheless, samples of process 1 (0.02/early/MM), process 6 (0.005/late/MM) and process 8 (0.005/late/LB) showed elevated skews and widths compared to the remaining processes despite the low expression of the oxygen limitation marker (TagRFP657 measured at 660/20 nm) during the lag-phase. The CV was quite similar in all the processes (Figure 9C).

The general location of the samples in the score plot only changed marginally in early exponential growth phase. While process 6 (0.005/late/MM) and process 8 (0.005/late/LB) kept their high level of population heterogeneity due to high skew and width, process 1 (0.02/early/MM) began to shift to the left-hand side towards higher mean and mode values (Figure 9D).

Upon entering the late exponential growth phase, this left shift applied to samples of other processes as well. While process 1 (0.02/early/MM) and process 6 (0.005/late/MM) showed the highest levels of mean and mode as well as skew and width, process 2 (0.005/earl/MM), process 5 (0.02/late/MM), process 7 (0.02/late/LB) and process 10 (0.0125/mid/MM) had moderate mean and mode values combined with a rather low level of population heterogeneity. Samples of the remaining processes, mainly compromising processes pre-grown in a complex medium, did not change their location in the score plot, indicating only minor oxygen limitation in these processes (Figure 9E).

Interestingly, the transition to stationary phase upregulated the expression of the oxygen limitation marker in additional processes. Now, samples of all processes except for process 4 (0.005/early/LB), process 8 (0.005/late/LB) and process 9 (0.0125/mid/LB) were located on the upper-left corner (negative PC_1_ and positive PC_2_ values), therefore, with high mean and mode values but less tendencies of population heterogeneity (Figure 9F).

## 4. Discussion

The process design of novel biotechnological production processes strives for providing robust and high-yield bioprocesses to meet the increasing demands of bio-based goods. While high yields are often realised by metabolic engineering and genetic modifications of the production strains [37,38], designing a robust process appears to be more challenging. Robustness in microbial production processes refer to the ability of the producing strain to maintain the production performance under diverse environmental conditions, whereas tolerance is part of a robust process but is only associated with growth-related parameters [39]. Both can be affected by a variety of factors such as the bioreactor design, the process control and also the pre-culture conditions [40]. In this study, we examined the influence of varying pre-culture conditions on the phenotypic expression and level of population heterogeneity in growth, general stress response and oxygen limitation response in the subsequent main batch culture by applying the *E. coli* triple reporter strain G7_BL21(DE3)_ [11]. Such a study has, to our knowledge, not been conducted before.

The evaluation of physiology on population and single-cell level in batch cultures of the *E. coli* triple reporter strain with principal component analysis revealed interesting trends and correlations regarding specific pre-culture conditions on the phenotypic development of growth, general stress response and oxygen limitation as well as the extent of differences in these expressions. Despite the use of an oxygen limitation marker, it is important to stress that the dissolved oxygen levels during cultivations in the stirred-tank bioreactor never decreased to below 20%. This is a crucial prerequisite for the use of reporter strains as the corresponding fluorescent proteins require oxygen for the correct maturation and thus the exhibition of fluorescence [41,42]. Additionally, it was reported that the *narGHIJ* operon was already activated during bioprocesses before anoxic conditions were reached [17].

Connecting the pattern found during the analysis of the population level response to the conditions in the pre-culture, as expected, all varied characteristics had an influence. Consequently, when aiming for a high space–time yield of biomass while avoiding by-product formation, the culture should be inoculated with cells from early exponential growth phase and with high optical density using the same medium in pre- and main cultures. These findings are indeed not new, but instead consistent with general practice and validate the reliability of the data analysis framework.

For the growth marker EmGFP expressed, together with the ribosomal promoter *rrnB*, the principal component analysis emphasised the impact of pre-culture conditions on the subsequent cultivation in a stirred-tank bioreactor regarding the growth levels (mean and mode) as well as the occurrence of population heterogeneity (CV, skew, width). The choice of cultivation media for the pre-cultures had an especially major impact. It was revealed that cells derived from pre-cultivation in minimal medium showed higher tendencies of population heterogeneity in the subsequent batch processes in the stirred-tank bioreactor, whereas cells grown in a complex medium during pre-cultivation expressed higher fluorescence levels and thus stronger growth during the batch processes. Additionally, the latter mentioned processes particularly showed higher mean and mode values in most phases when inoculated with higher cell densities. Moreover, the inoculation of higher optical densities of cells grown in a complex medium reduced the lag-phase, accelerated the onset of cell growth in the new environment and led to even higher fluorescence values, respectively, during the subsequent batch process. The fact that higher inoculation densities enhance initial growth and viability was already stated by others [43,44]. Generally, differences in the level of heterogeneity and fluorescence intensity were most pronounced for processes, which were inoculated with fresh or mid-aged cells from pre-cultivation, whereas cells derived from late phases during pre-cultivation showed comparably less deviations.

Interestingly, cells derived from minimal medium during pre-cultivation experienced a stronger oxygen limitation and were more sensitive regarding lower dissolved oxygen levels, according to the fluorescence data and the corresponding principal component analysis. This might explain the lower expression of EmGFP. Further correlations between pre-culture conditions and the level of population heterogeneity in stirred-tank bioreactor processes were not found. This might be due to the comparably low expression levels of this marker so that the effects of pre-culture conditions might be dimmed [11].

Regarding the general stress response of the cells, again, the key factor for differences in population heterogeneity in general stress response levels was the applied pre-culture medium. Therefore, the inoculation of cells derived from a complex medium during the pre-cultivation at a late stage and with high initial optical densities promoted the general stress response in the subsequent batch process the most. In particular, the sudden switch from a nutrient-rich complex medium to the limiting minimal medium could have perturbated the cells directly from the beginning of the batch process, leading to higher expression levels and higher levels of population heterogeneity in comparison to cultures pre-grown in minimal medium. Additionally, *E. coli* cells often show a low expression of the general stress response marker *rpoS* during the exponential growth phase, while this substrate-related marker increases strongly when entering the stationary phase [36]. Interestingly, an early harvest time of pre-culture cells grown in complex medium made the occurrence of population heterogeneity more likely at process start. On the other hand, a late harvest of the cells led to higher general stress response levels. However, population heterogeneity levels were more pronounced in processes inoculated with a low initial optical density, whereas an initial high optical density induced higher expression levels at the end of the process, especially for the processes inoculated with cells grown in complex medium. Nevertheless, it is surprising that despite the higher general stress response levels, cell growth was still unaffected.

As a summary regarding the ideal process design in terms of tolerance, cells grown in complex medium in pre-cultivation, preferably inoculated with fresh cells at a high optical density, performed slightly better in the subsequent batch process than cells derived from minimal medium, according to the fluorescence data. In terms of reproducibility, lower levels of population heterogeneity are desired [45]. However, there were no clear trends observed at which exact pre-culture conditions particularly enhanced or diminished the occurrence of subpopulations. Also, the question arises whether the here recognised tendencies of subpopulation formation were induced by external factors such as pre-culture medium or by intrinsic factors such as differential gene expressions within the cells [2,39]. The robustness cannot comprehensively be evaluated due to the missing link to product formation in this strain, but will be the focus of upcoming research.

Generally, the principal component analysis successfully supported a faster comprehension of this larger dataset and revealed trends and correlations, as this tool was also used with great success in other studies [46,47]. The here used principal component analysis could be further improved by the implementation of clusters based on the Euclidean distance in the score plot. However, it is impossible to manually perform this for large datasets. Therefore, in future studies, automated methods should be established, for instance, by adapting a method for the gating of high-dimensional fluorescence data [48]. As a benefit, biased gates and clusters can be avoided and irregular shapes could be drawn, which would then lead to a clearer visualisation of the clusters.

## Figures and Tables

**Table 1:** Overview of the experimental design varying optical density at 600 nm at inoculation of the bioreactor (OD_600_), pre-culture medium (minimal medium (MM) [35]; lysogeny broth (LB)) and pre-culture harvest time point in exponential growth phase.

Process	Inoculation OD_600_	Pre-Culture Harvest in Exponential Growth Phase	Pre-Culture Medium
1	0.02	Early (6 h)	MM
2	0.005	Early (6 h)	MM
3	0.02	Early (3.5 h)	LB
4	0.005	Early (3.5 h)	LB
5	0.02	Late (9 h)	MM
6	0.005	Late (9 h)	MM
7	0.02	Late (7 h)	LB
8	0.005	Late (7 h)	LB
9	0.0125	Mid (5 h)	LB
10	0.0125	Mid (7 h)	MM

## Data Availability

The data presented in this study are available on request from the corresponding author. Process data of all stirred-tank bioreactor cultivations are provided in an Excel sheet as supplementary material. Data of the principal component analysis on population level and single-cell level will be provided on request.

## Supplementary Materials

The following supporting information can be downloaded at: https://www.mdpi.com/article/10.3390/microorganisms11071763/s1, Figure S1: Optical density at 600 nm in batch cultures with the *E. coli* triple reporter strain G7_BL21(DE3)_ in shake flasks with lysogeny broth (LB) (A) and minimal medium according to Riesenberg [35](B) at 37 °C and 150 rpm. Biological triplicate cultures were inoculated with a single colony from agar plates with the same medium as used in the pre-culture. Blue circles indicate the time points in early, mid and late exponential growth phase at which samples were withdrawn from the pre-culture to inoculate main cultures in bioreactors. The grey square visualises the exponential growth phase; Figure S2: Fluorescence distributions of the *E. coli* triple reporter strain G7_BL21(DE3)_ measured with flow cytometry and harvested in early, mid and late exponential phase from shake flask cultures with lysogeny broth (LB) (A) and minimal medium according to Riesenberg [35] incubated at 37 °C and 150 rpm. Fluorescence evaluated *rrnB*-EmGFP expression, which correlated to single-cell growth, *rpoS*-mStrawberry expression related to general stress response of single cells and *nar-*TagRFP657 expression related to oxygen availability of single cells; Figure S3: Coefficient of variance against mean fluorescence for distributions of single-cell growth (*rrnB*-EmGFP expression, A), general stress response (*rpoS*-mStrawberry expression, B) and oxygen availability (*nar-*TagRFP657 expression, C) of single cells in shake flask cultures of the *E. coli* triple reporter strain G7_BL21(DE3)_ with LB medium (green diamonds) and minimal medium (red circles) according to Riesenberg [35] incubated at 37 °C and 150 rpm; Figure S4: Yield coefficients and carbon balance for biomass (X, blue), acetate (Ac, red), CO_2_ (green), formate (For, purple), malate (Mal, light blue), lactate (Lac, orange) and succinate (Suc, grey) on glucose in batch cultivations in stirred-tank bioreactors of the *E. coli* triple reporter strain G7_BL21(DE3)_ in minimal medium with glucose as carbon source varying optical density at 600 nm for inoculation of the bioreactor (0.005, 0.02 or 0.0125), harvest time point in exponential phase of the pre-culture (early, mid or late) and medium used for the pre-culture (LB: lysogeny broth and MM: minimal medium according to Riesenberg [35]). The red line marks 100% of carbon recovery; Figure S5: Schematic overview of the screening procedure of cellular physiologies by integrated reporter molecules.
