# Supplementary material for: Influence of Varying Pre-Culture Conditions on the Level of Population Heterogeneity in Batch Cultures with an Escherichia coli Triple Reporter Strain
Source: Microorganisms. 2023 Jul 6;11(7):1763. doi: 10.3390/microorganisms11071763 (PMC10384452; doi:10.3390/microorganisms11071763)
Supplement: Supplementary file 1 [file microorganisms-11-01763-s001.zip › Supplementary_material_microorganisms.pdf]

Supplementary material

Evaluation of the influence of pre-culture conditions on the level of population heterogeneity in batch cultures with *Escherichia coli*

Manh Dat Hoang[1], Sophi Riessner[1], Jose Enrique Oropeza Vargas[1], Nikolas von den Eichen[1], Anna-Lena Heins[1]

[1] Chair of Biochemical Engineering, TUM School of Engineering and Design, Technical University of Munich, 85748 Garching, Germany; dat.hoang@tum.de (M.D.H.), ga87bok@mytum.de (S.R.), enrique.oropeza@tum.de (J.E.O.V.), nikolas.eichen@tum.de (N.v.d.E.), anna-lena.heins@tum.de (A-L.H.)

* Correspondence: anna-lena.heins@tum.de; Phone: +49-89-289-15729

Supplementary material I – pre-cultivation in shake flasks

To evaluate the course of pre-cultures in lysogeny broth (LB) and minimal medium according to Riesenberg (Riesenberg et al. 1991), respective cultivations in shake flasks were performed. Based on the acquired data, time points in early, mid and late exponential growth phase for harvest of the cells for inoculation of the main culture were chosen.

Materials and methods

Single colonies from minimal medium according to Riesenberg (Riesenberg et al. 1991) (MM) or lysogeny broth (LB) agar plates of the E. coli triple-reporter strain $G7_{BL21(DE3)}$ were used to inoculate biological triplicates of 50 mL MM (Riesenberg et al. 1991) or LB cultures in 500 mL baffled shake flasks. The flasks were shaken at 150 rpm and 37 °C. Meanwhile, cultures were regularly sampled for optical density at 600 nm (OD_{600}) and flow cytometry (FC) analysis until they reached stationary phase.

Results

Population level physiology

Cultures in shake flasks with minimal (figure S1, A) and LB medium (figure S1 B) both exhibited typical growth behaviour of *Escherichia coli*.

In LB medium (figure S1, A) the *E.coli* triple reporter strain $G7_{BL21(DE3)}$ showed a lag-phase respectively phase of slow growth of about two hours, where after the optical density at 600 nm started to slightly rise in a transitional phase. After around three hours, the cells started to growth exponentially with a growth rate of 0.984 ± 0.025. This phase lasted for around four hours, when the culture gradually reached stationary phase with a maximal optical density at 600 nm of 4.39 ± 0.01 after 8 h of culture

Cultures in minimal medium (figure S1, B) expectedly exhibited a slightly longer lag-phase after inoculation before they started growing. First after around five hours growth was visible on population level. After a minor transition phase, cultures started to grow exponentially until around 10 hours after inoculation. The growth rate in this phase was determined to be 0.948 ± 0.054 and led to a final optical density at 600 nm after 10.2 hours of 4.93 ± 0.23 in stationary phase.

Since the cells for inoculation of the main culture should be harvested in early, mid and late exponential growth phase, based on the growth curves the transfer of cells from cultures grown on LB medium was chosen after 3.5 h, 5 h and 7 h. Equivalently, cultures grown on minimal medium were harvested after 6 h, 7 h and 9 h of culture.

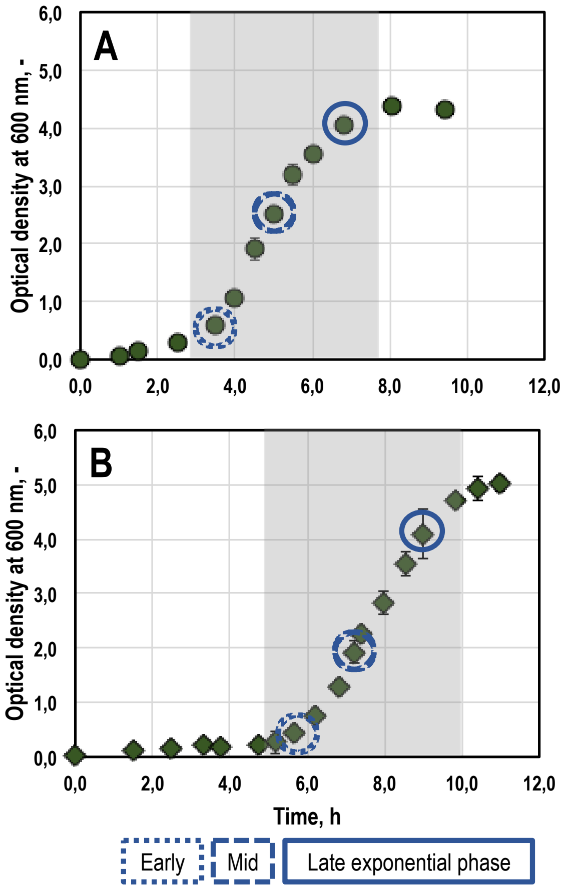

Figure S1. Optical density at 600 nm in batch cultures with the *E. coli* triple reporter strain G7BL21(DE3) in shake flasks with lysogeny broth (LB) (A) and minimal medium according to Riesenberg (Riesenberg et al. 1991) (B) at 37 °C and 150 rpm. Biological triplicate cultures were inoculated with a single colony from agar plates with the same medium as used in the pre-culture. Blue circles indicate the time points in early-, mid- and late exponential growth phase at which samples were withdrawn from the pre-culture to inoculate main cultures in bioreactors. The grey square visualizes the exponential growth phase

Single cell physiology

To quantify the level of heterogeneity in the pre-culture in shake flasks prior to inoculation of the main culture in the bioreactor and investigate whether time points in exponential growth phase on population level match with the behaviour on single cell level, samples were also taken for flow cytometry analysis of fluorescence of the *E. coli* triple reporter strain G7 $_{BL21(DE3)}$. In this way, single cell growth could be followed by expression of EmGFP together with the ribosomal promoter *rrnB*, general stress response of single cells by expression of mStrawberry together with expression of the gene for the sigma factor *rpoS* and expression of *nar*-TagRFP657 to gain information about oxygen limitation of single cells.

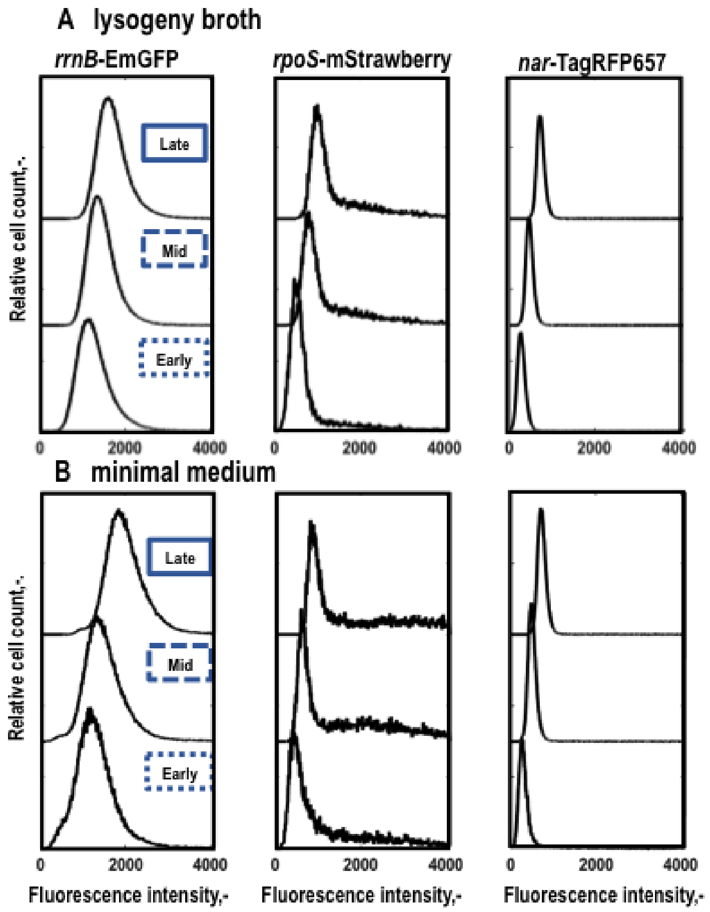

Figure S2. Fluorescence distributions of the *E. coli* triple reporter strain G7$_{BL21(DE3)}$ measured with flow cytometry and harvested in early, mid and late exponential phase from shake flask cultures with lysogeny broth (LB) (A) and minimal medium according to Riesenberg (Riesenberg et al. 1991) incubated at 37 °C and 150 rpm. Fluorescence was evaluated of *rrnB*-

EmGFP expression which correlated to single cell growth, *rpoS*-mStrawberry expression related to general stress response of single cells and *nar*-TagRFP657 expression related to oxygen availability of single cells

Single cell growth. Distributions for single cell growth followed by expression of EmGFP-*rrnB* exhibited a similar trend in both media with increasing overall and mean fluorescence in the course of the exponential growth phase (figure S2, left and S3A), which was expected due to the characteristics of the marker [REF]. The general shape of the distributions resembled a normal distribution with a minor right skew for cultures in both media. For cultures in LB medium the distributions were apparently narrower compared to cultures in minimal medium (figure S2A and B), however for both cultures the width of the distributions remained about the same in early, mid and late exponential growth phase. This is also confirmed by the coefficient of variance (figure S3A) that decreased for cultures in both media with increasing mean fluorescence levels. However, for cultures in LB medium this decrease was linear whereas for cultures in minimal medium the decrease was stronger between mid and late exponential phase than between early and mid exponential phase, pointing towards a stronger influence of heterogeneity towards the end of the exponential growth phase.

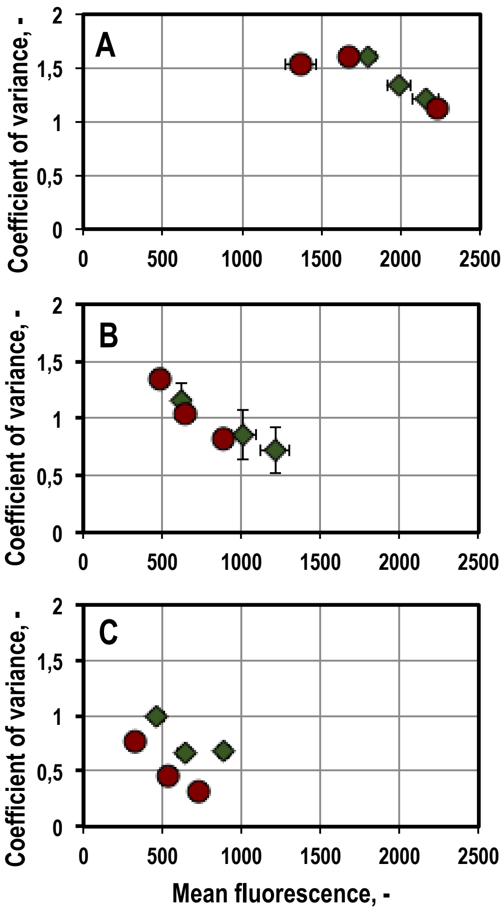

Figure S3. Coefficient of variance against mean fluorescence for distributions of single cell growth (*rrnB*-EmGFP expression, A), general stress response (*rpoS*-mStrawberry expression, B) and oxygen availability (*nar*-TagRFP657 expression, C) of single cells in shake flask cultures of the *E. coli* triple reporter strain G7$_{\mathrm{BL21(DE3)}}$ with LB medium (green diamonds) and minimal medium (red circles) according to Riesenberg (Riesenberg et al. 1991) incubated at 37 °C and 150 rpm

General stress response of single cells. The distributions for the general stress response of single cells that correlate with the expression pattern of mStrawberry-*rpoS*, were generally narrow concerning the main population but exhibited some noise in higher fluorescence channels (figure S2, middle) This noise was stronger for cultures in minimal medium compared to LB medium, which was also visible in the higher coefficient of variance for this culture (figure S3B) and might point towards the appearance of a minor subpopulation of cells with elevated stress levels in both cultures. This subpopulation apparently increased in the course of the exponential growth phase. However, comparing mean fluorescence levels (figure S3B) cells in LB medium seem to reach a slightly higher level of stress at the end of the

exponential growth phase than cells cultured in minimal medium. Additionally, stress levels significantly rose from mid exponential phase on, whereas for cells cultured in minimal medium exhibited a constant rise through out the culture. Probably the nutrient situation in LB medium changed more abruptly in comparison to minimal medium cultures.

Oxygen limitation of single cells. Oxygen limitation that was measured by fluorescence distributions of TagRFP657 expressed together with the *nar*-operon, could be observed in both cultures starting from mid exponential growth phase (figure S2, right and figure S3C). Distributions in both media were narrow and resembled a normal distribution that got narrower and thereby less heterogeneous as the culture progresses, especially for cultures in minimal medium (figure S3C).

Since the distributions in early, mid and late exponential phase in both media were generally similar and matched the expected pattern due to the characteristics of the markers of the triple reporter strain, it was concluded that with the chosen harvest points of the pre-culture early, mid and late exponential growth phase could also be covered on single cell level

Supplementary material II – Yields and carbon balances

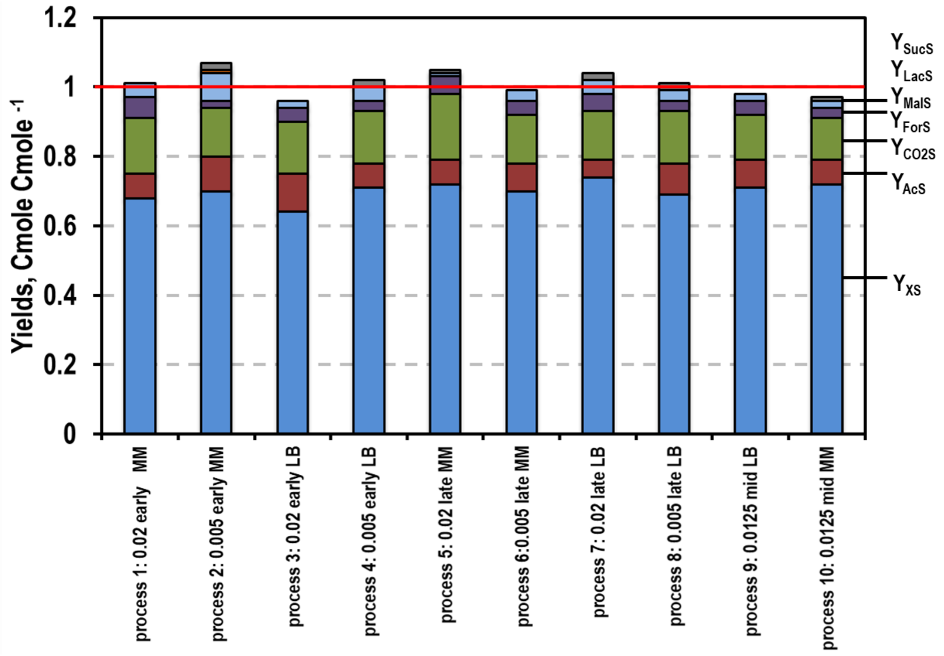

Figure S4. Yield coefficients and carbon balance for biomass (X, blue), acetate (Ac, red), CO_2 (green), formate (For, purple), malate (Mal, light blue), lactate (Lac, orange) and succinate (Suc, grey) on glucose in batch cultivations in stirred-tank bioreactors of the *E. coli* triple reporter strain $G7_{BL21(DE3)}$ on minimal medium with glucose as carbon source varying optical density at 600 nm for inoculation of the bioreactor (0.005, 0.02 or 0.0125), harvest time point in exponential phase of the pre-culture (early, mid or late) and medium used for the pre-culture (LB: lysogeny broth and MM: minimal medium according to (Riesenberg et al. 1991)). The red line marks 100% of carbon recovery

Supplementary material III – Principal concept of reporter strains for monitoring of cellular physiologies

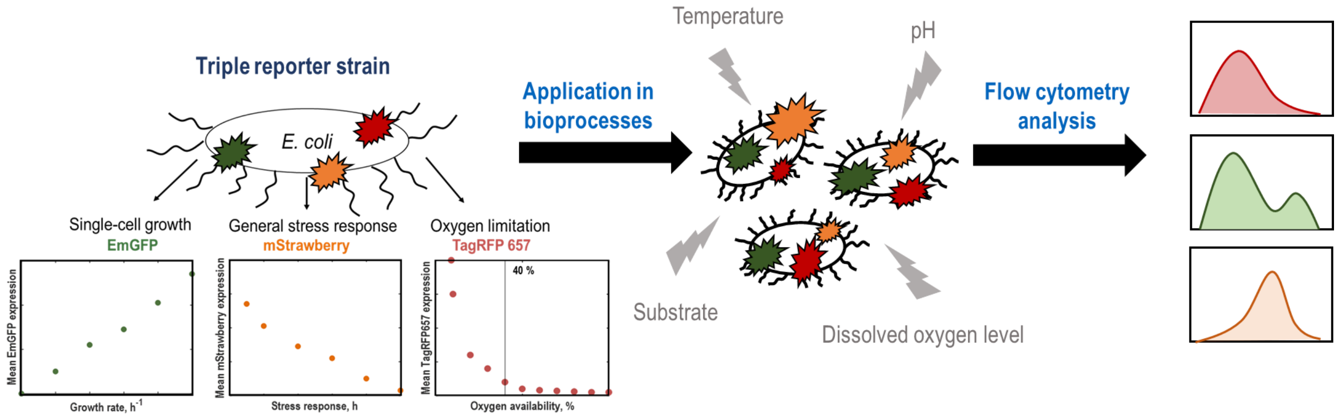

Figure S5. Schematic overview of the screening procedure of cellular physiologies by integrated reporter molecules.

Supplementary material IV – plots for general physiology of all bioprocesses

Process 1 (0.02, early, MM)

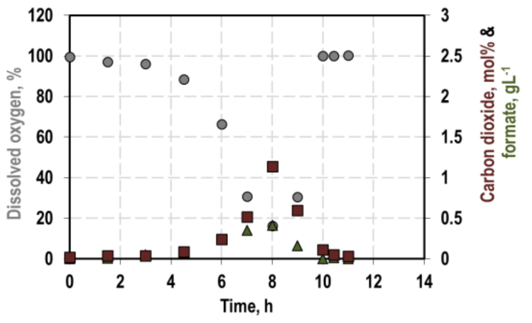

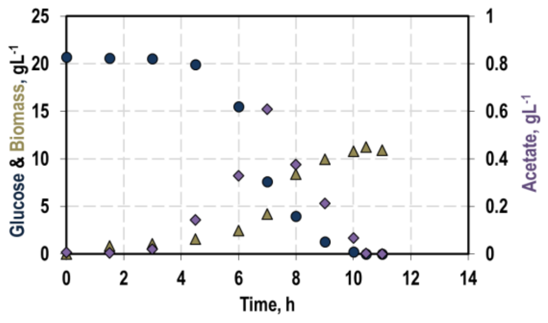

Process 2 (0.005, early, MM)

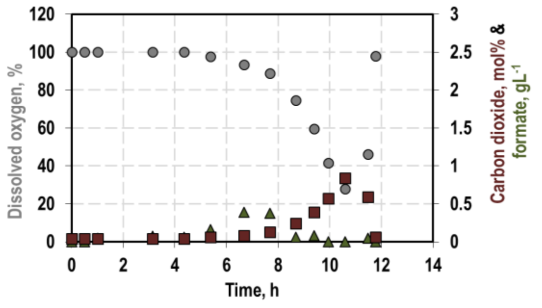

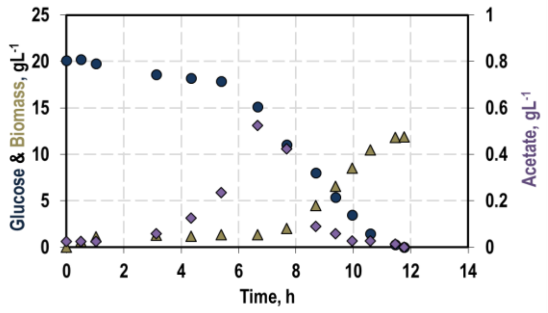

Process 3 (0.02, early, LB)

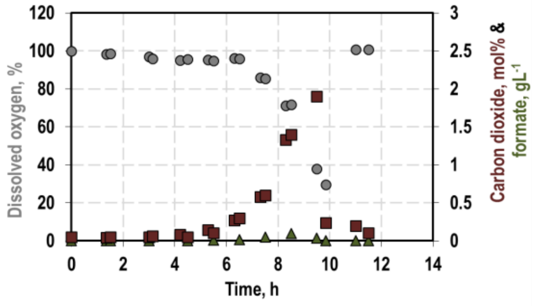

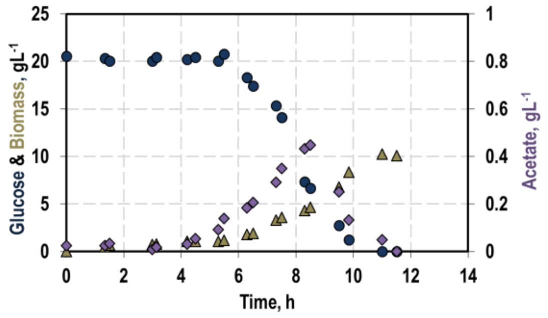

Process 4 (0.005, early, LB)

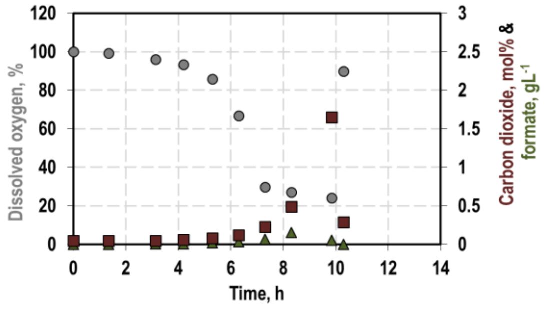

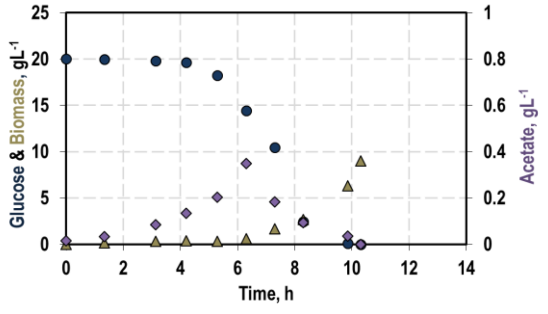

Process 5 (0.02, late, MM)

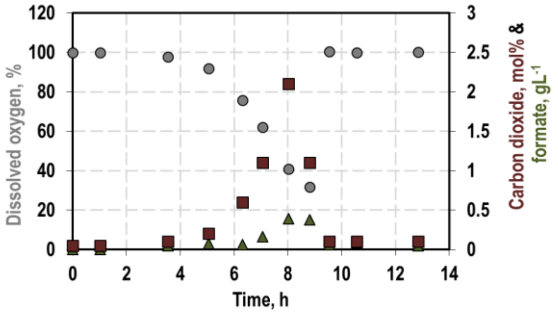

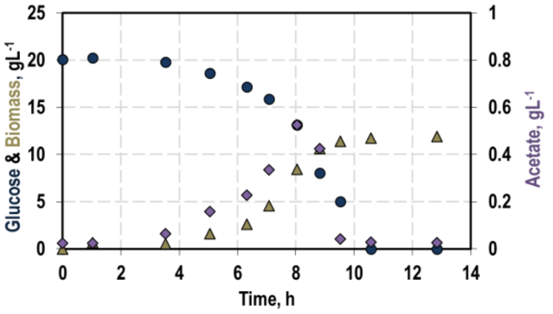

Process 6 (0.005, late, MM)

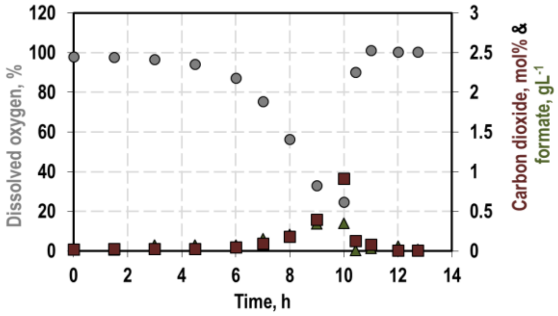

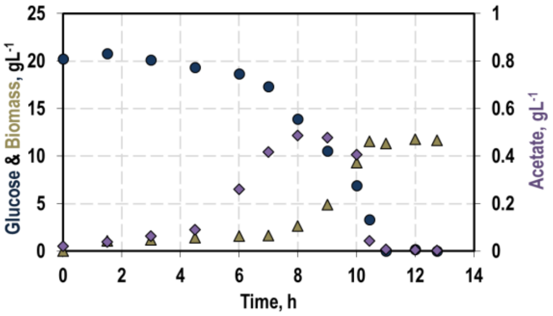

Process 7 (0.02, late, LB)

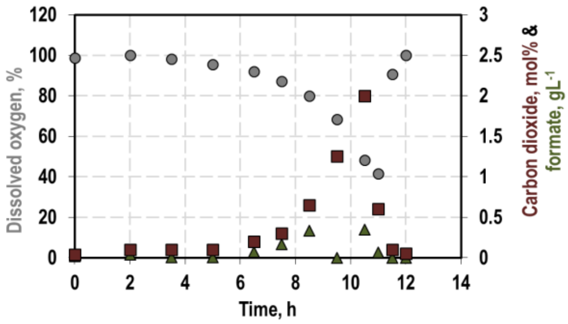

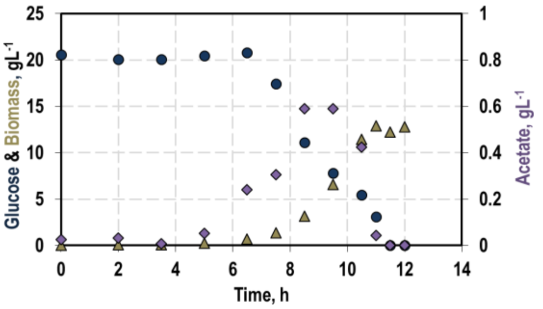

Process 8 (0.005, late, LB)

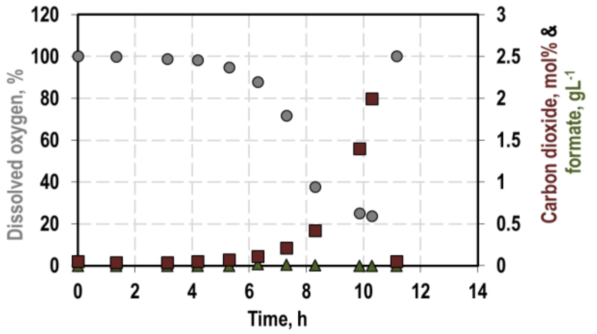

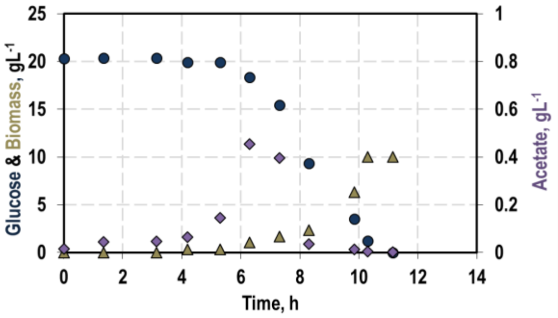

Process 9 (0.0125, mid, LB)

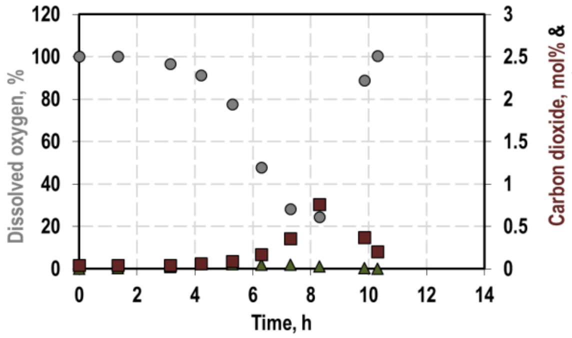

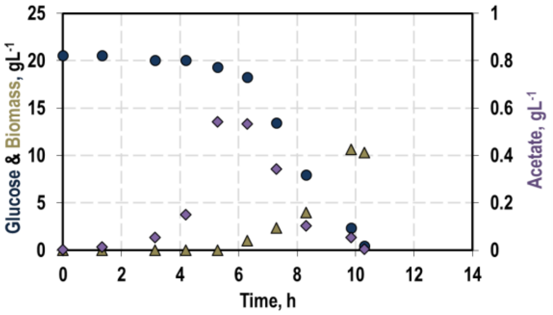

Process 10 (0.0125, mid, MM)

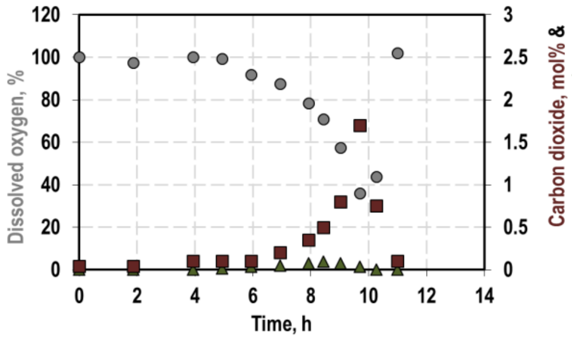

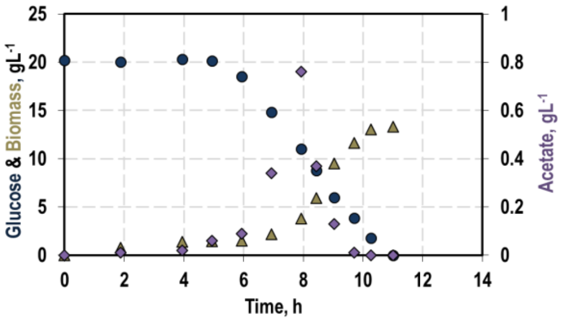

Supplementary material V – plots for general trends of single cell growth of all bioprocesses

Process 1 (0.02, early, MM)

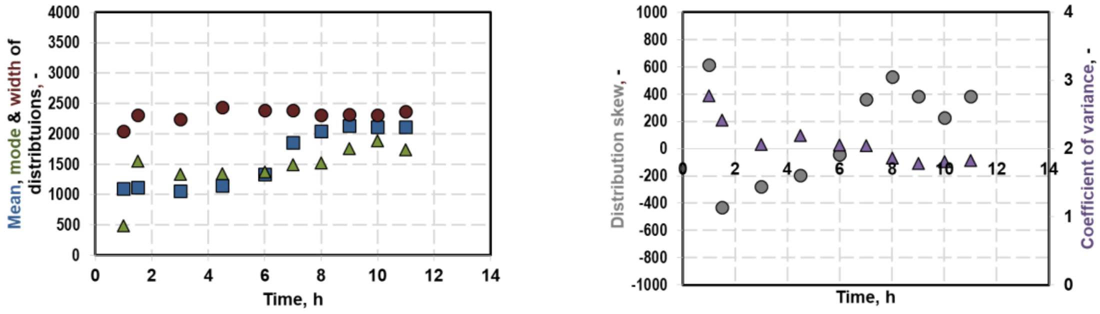

Process 2 (0.005, early, MM)

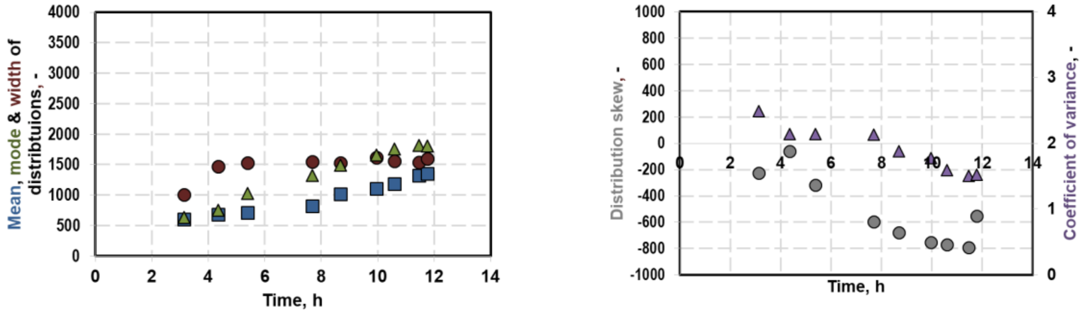

Process 3 (0.02, early, LB)

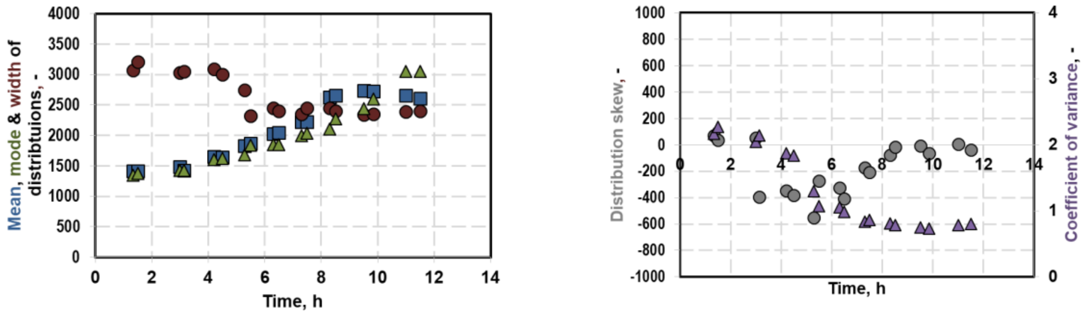

Process 4 (0.005, early, LB)

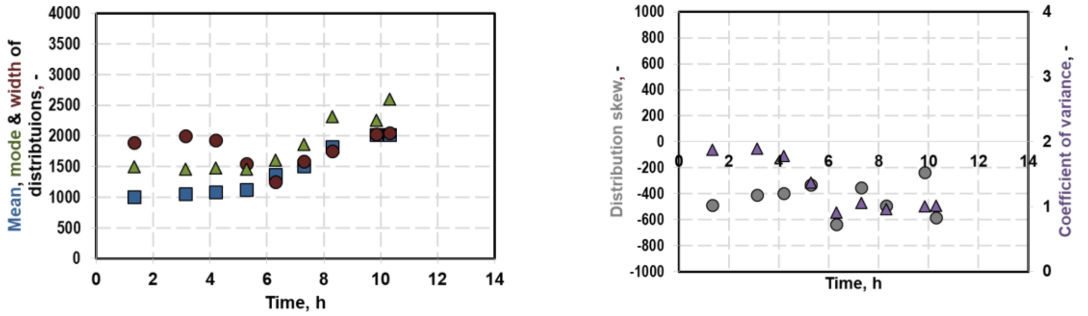

Process 5 (0.02, late, MM)

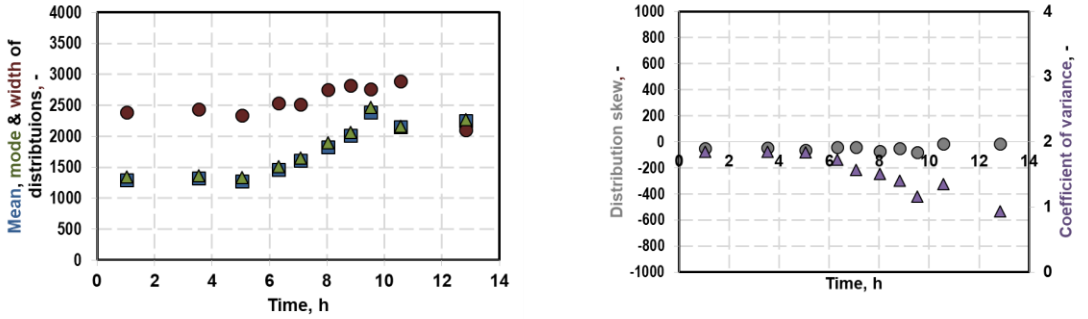

Process 6 (0.005, late, MM)

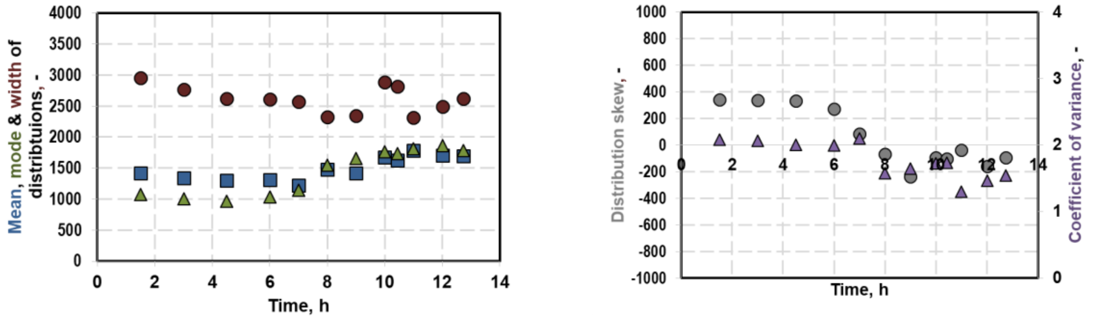

Process 7 (0.02, late, LB)

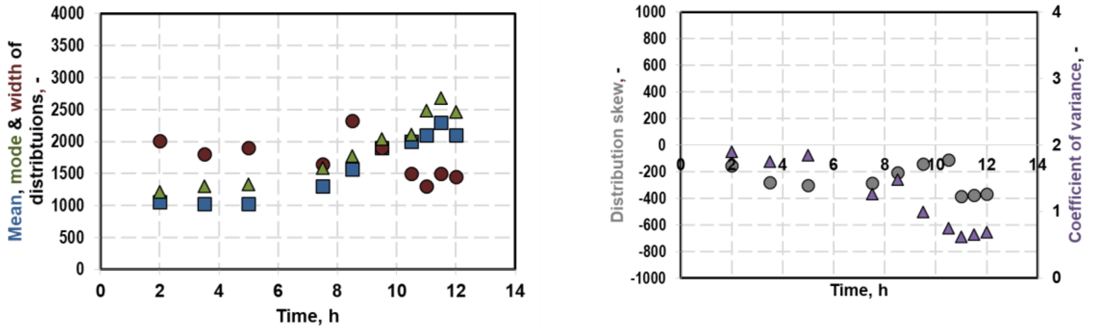

Process 8 (0.005, late, LB)

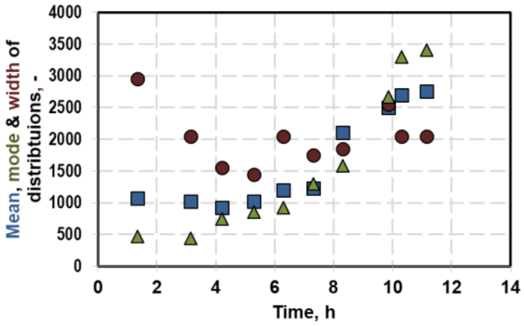

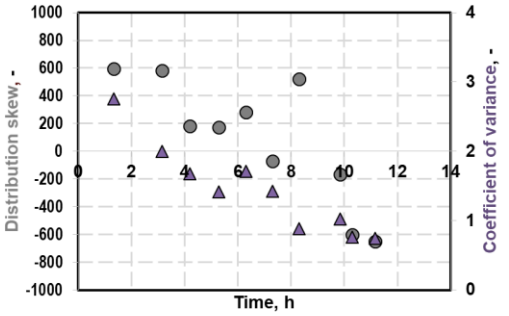

Process 9 (0.0125, mid, LB)

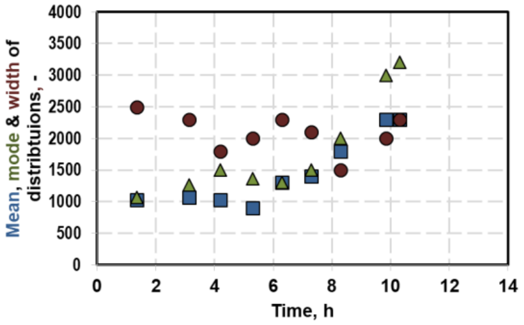

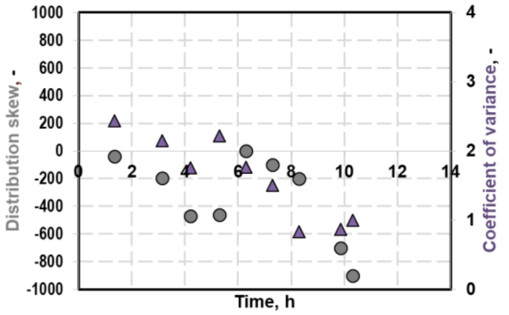

Process 10 (0.0125, mid, MM)

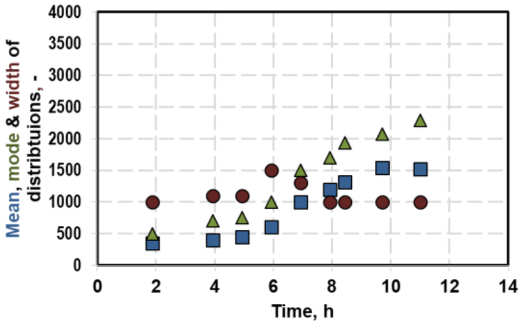

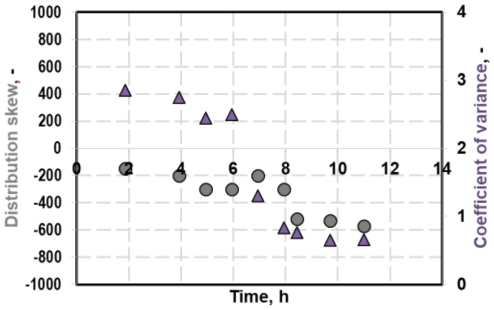

Supplementary material VI – plots for general trends of general stress response of single cells of all bioprocesses

Process 1 (0.02, early, MM)

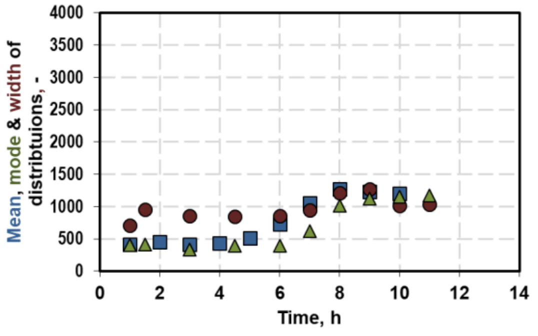

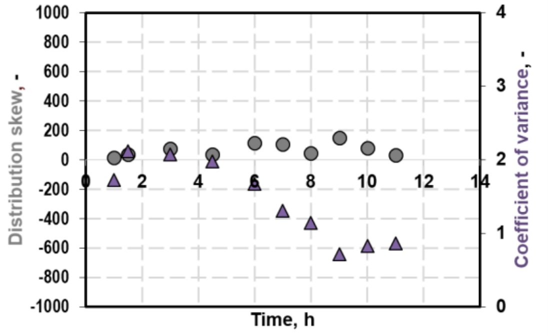

Process 2 (0.005, early, MM)

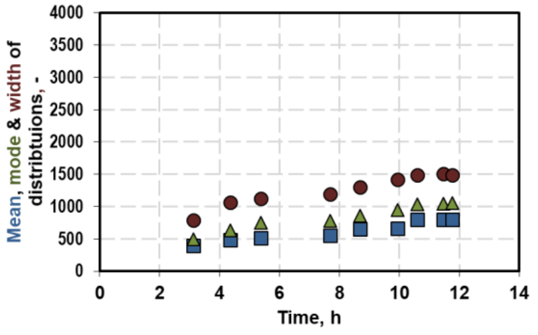

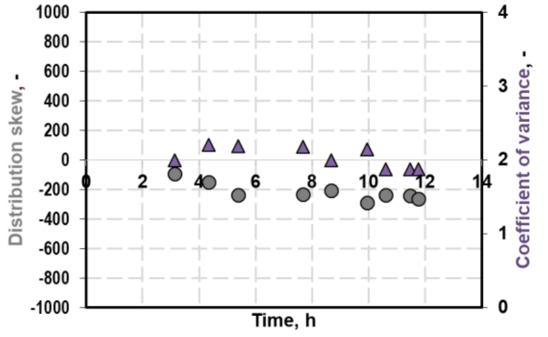

Process 3 (0.02, early, LB)

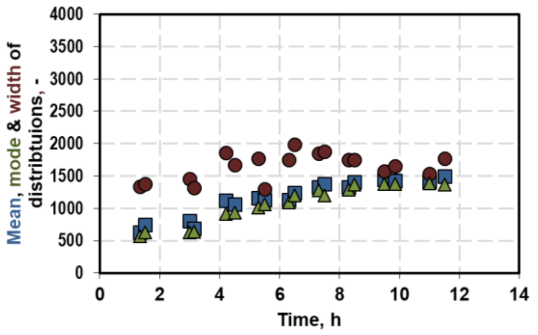

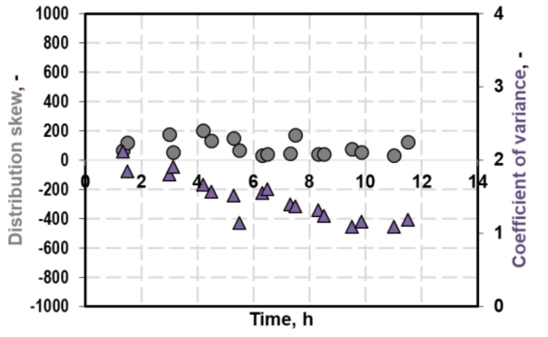

Process 4 (0.005, early, LB)

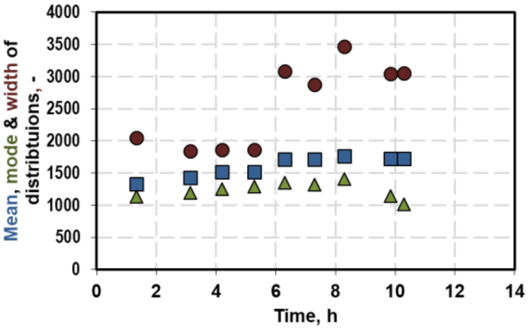

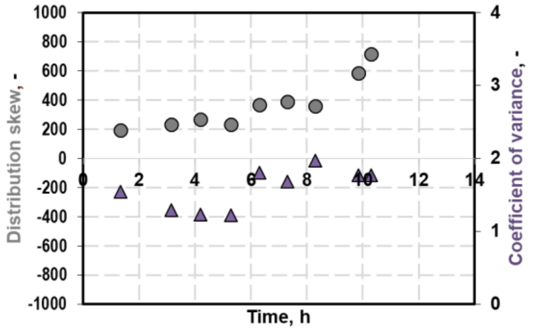

Process 5 (0.02, late, MM)

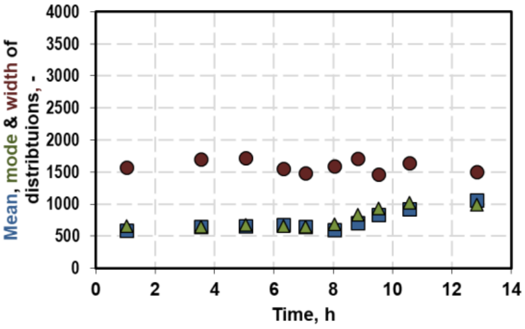

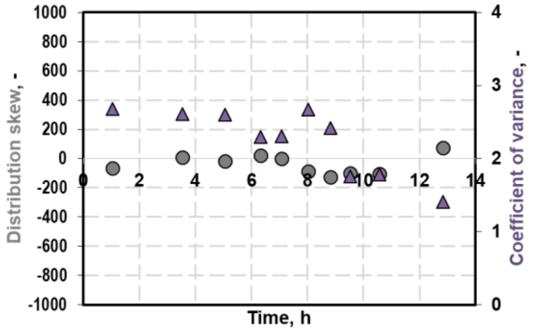

Process 6 (0.005, late, MM)

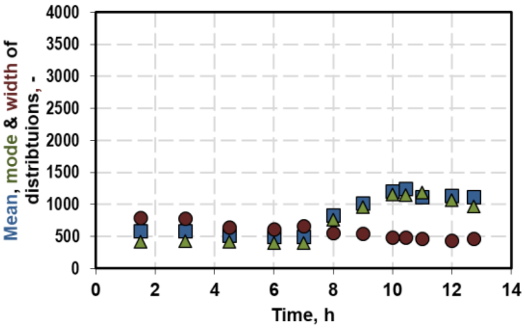

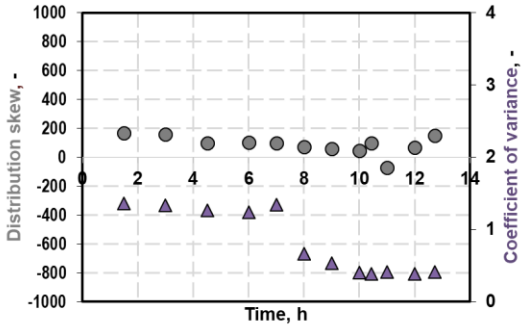

Process 7 (0.02, late, LB)

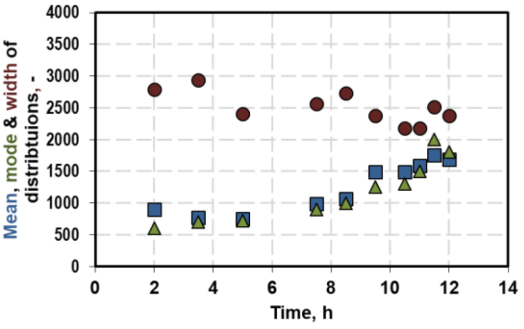

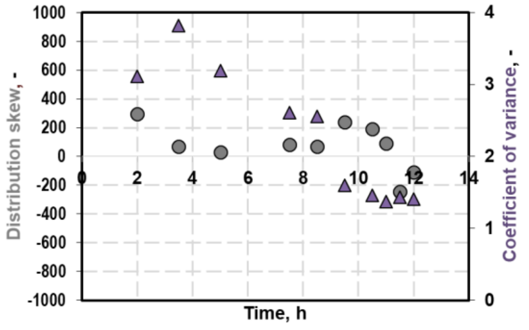

Process 8 (0.005, late, LB)

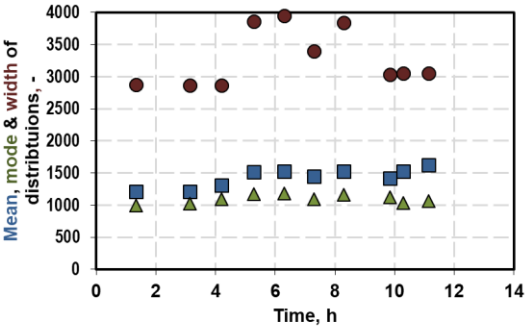

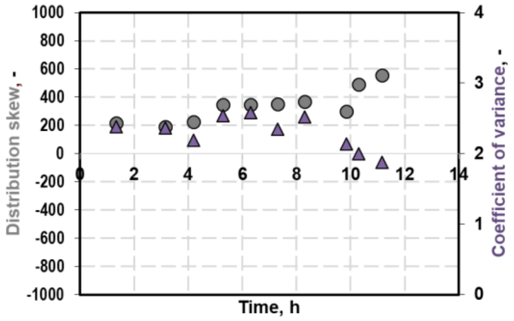

Process 9 (0.0125, mid, LB)

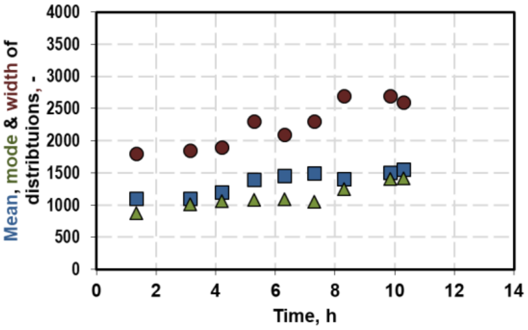

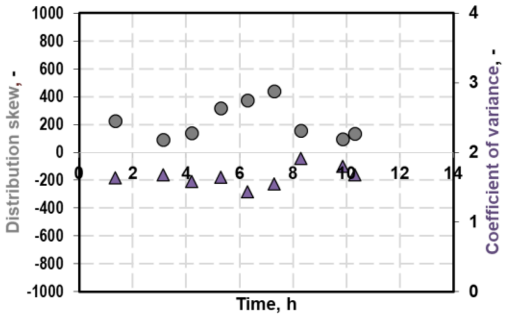

Process 10 (0.0125, mid, MM)

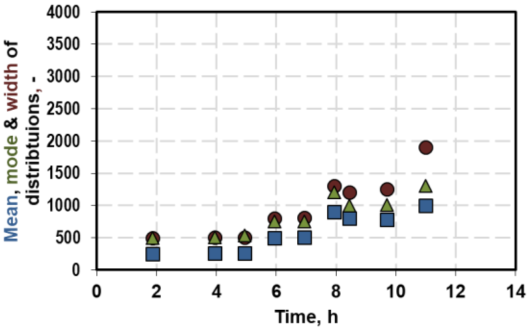

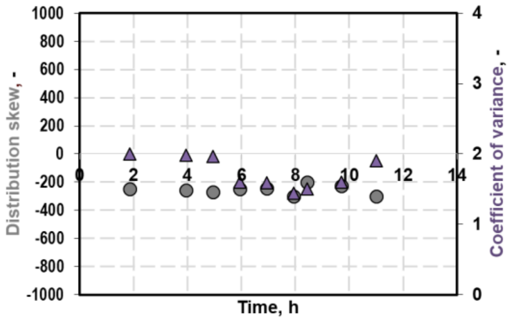

Supplementary material VII – plots for general trends of oxygen limitation of single cells of all bioprocesses

Process 1 (0.02, early, MM)

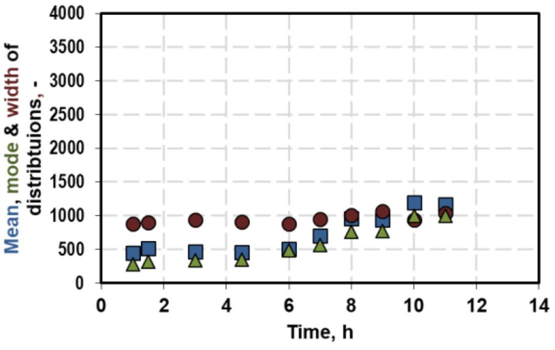

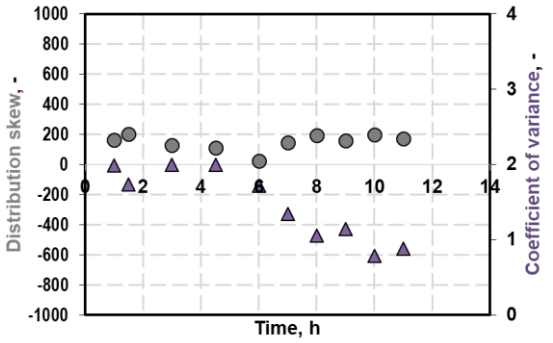

Process 2 (0.005, early, MM)

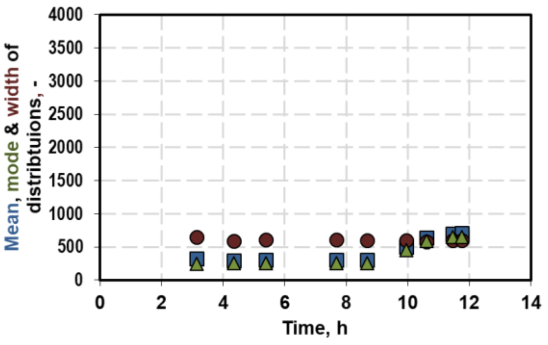

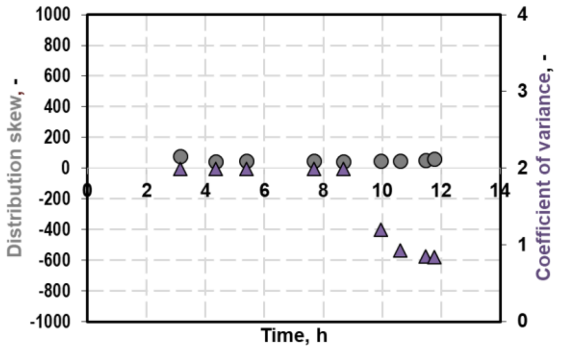

Process 3 (0.02, early, LB)

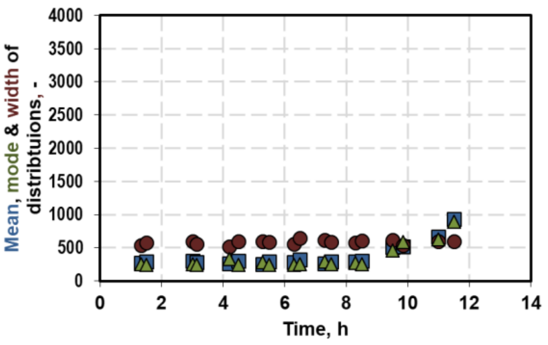

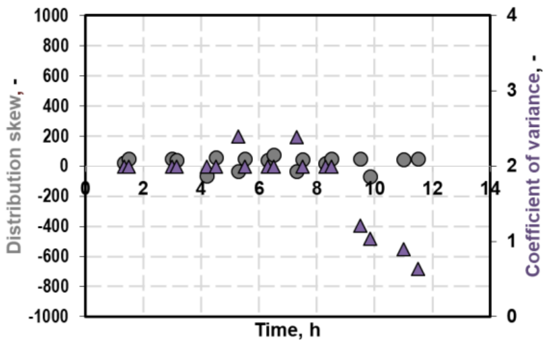

Process 4 (0.005, early, LB)

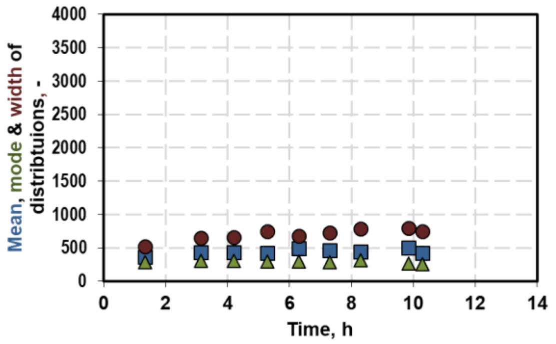

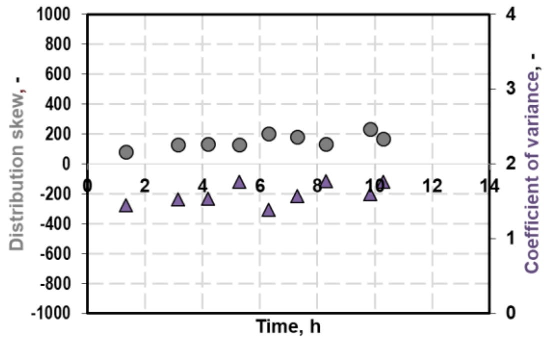

Process 5 (0.02, late, MM)

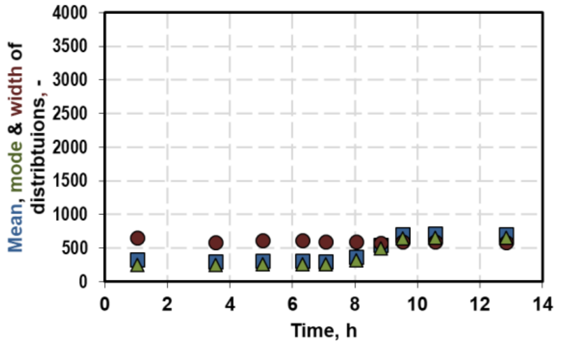

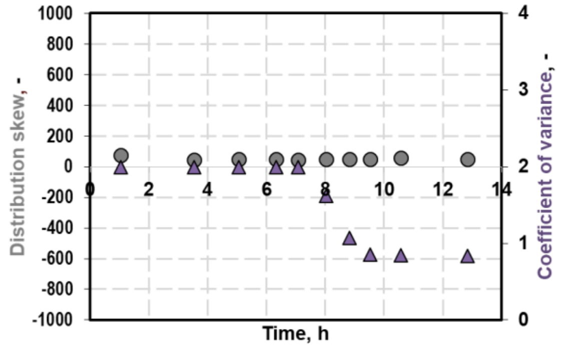

Process 6 (0.005, late, MM)

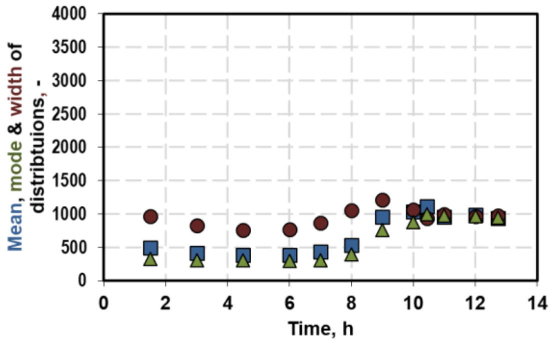

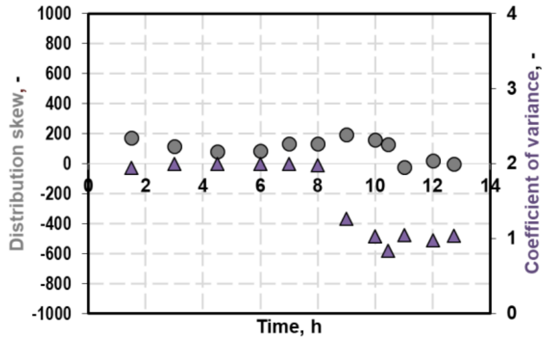

Process 7 (0.02, late, LB)

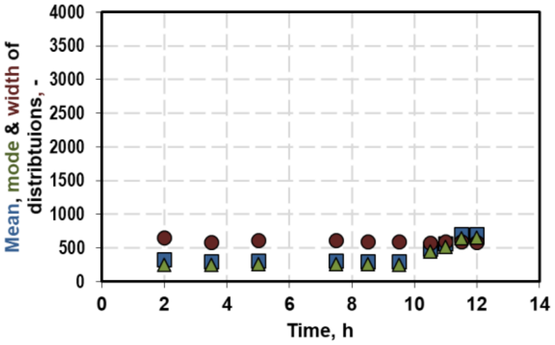

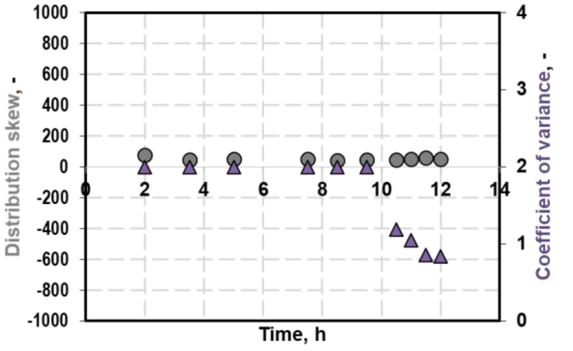

Process 8 (0.005, late, LB)

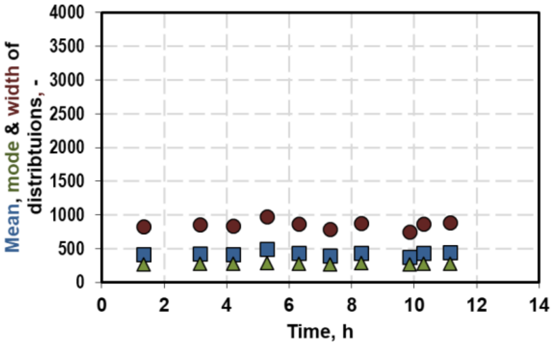

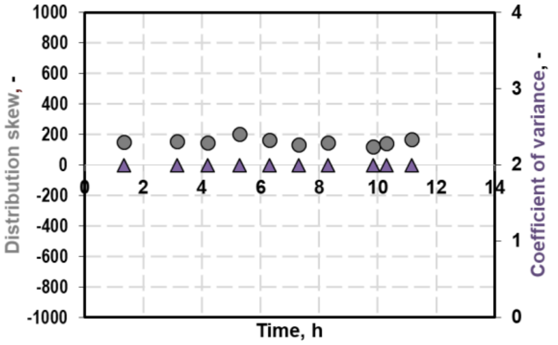

Process 9 (0.0125, mid, LB)

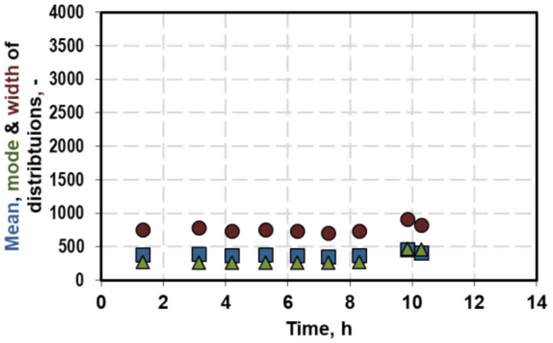

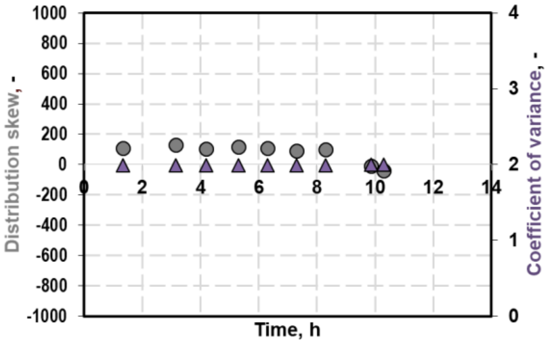

Process 10 (0.0125, mid, MM)

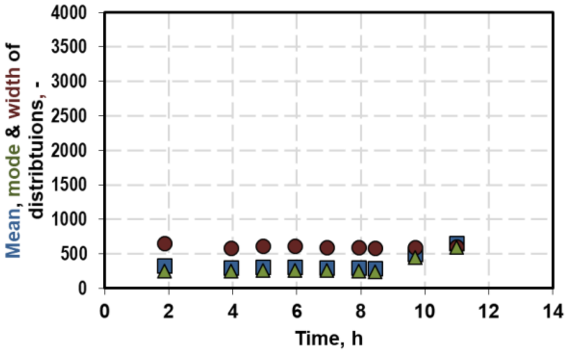

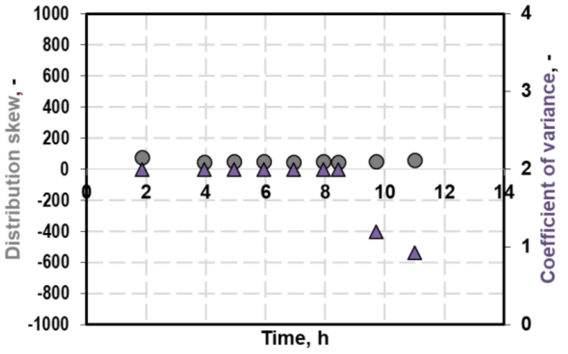

Reference

Riesenberg D, Schulz V, W.A. K, Pohl H-D, Korz D, Sanders EA, Roß A, Deckwer W-D (1991) High cell density cultivation of *Escherichia coli* at controlled specific growth rate. Journal of biotechnology 20:17-28
